# Structural insights into the function-modulating effects of nanobody binding to the integrin receptor α_M_β_2_

**DOI:** 10.1016/j.jbc.2022.102168

**Published:** 2022-06-20

**Authors:** Rasmus K. Jensen, Henrik Pedersen, Josefine Lorentzen, Nick Stub Laursen, Thomas Vorup-Jensen, Gregers Rom Andersen

**Affiliations:** 1 Department of Molecular Biology and Genetics, Aarhus University, Denmark; 2 Department of Biomedicine, Aarhus University, Denmark

**Keywords:** integrin, complement, CD11b/CD18, nanobody, crystallography, BLI, biolayer interferometry, BSA, bovine serum albumin, C3, component 3, CDR, complementarity-determining region, HP, headpiece, ICAM, intercellular adhesion molecule, I-EGF1, integrin epidermal growth factor 1, LA-1, leukadherin-1, MFI, mean fluorescence intensity, MIDAS, metal ion–dependent adhesion site, Nb, nanobody, PDB, Protein Data Bank, PSI, plexin–semaphorin–integrin, SEC, size-exclusion chromatography, SLE, systemic lupus erythematosus, SPR, surface plasmon resonance

## Abstract

The integrin receptor α_M_β_2_ mediates phagocytosis of complement-opsonized objects, adhesion to the extracellular matrix, and transendothelial migration of leukocytes. However, the mechanistic aspects of α_M_β_2_ signaling upon ligand binding are unclear. Here, we present the first atomic structure of the human α_M_β_2_ headpiece fragment in complex with the nanobody (Nb) hCD11bNb1 at a resolution of 3.2 Å. We show that the receptor headpiece adopts the closed conformation expected to exhibit low ligand affinity. The crystal structure indicates that in the R77H α_M_ variant, associated with systemic lupus erythematosus, the modified allosteric relationship between ligand binding and integrin outside–inside signaling is due to subtle conformational effects transmitted over a distance of 40 Å. Furthermore, we found the Nb binds to the αI domain of the α_M_ subunit in an Mg^2+^-independent manner with low nanomolar affinity. Biochemical and biophysical experiments with purified proteins demonstrated that the Nb acts as a competitive inhibitor through steric hindrance exerted on the thioester domain of complement component iC3b attempting to bind the α_M_ subunit. Surprisingly, we show that the Nb stimulates the interaction of cell-bound α_M_β_2_ with iC3b, suggesting that it may represent a novel high-affinity proteinaceous α_M_β_2_-specific agonist. Taken together, our data suggest that the iC3b–α_M_β_2_ complex may be more dynamic than predicted from the crystal structure of the core complex. We propose a model based on the conformational spectrum of the receptor to reconcile these observations regarding the functional consequences of hCD11bNb1 binding to α_M_β_2_.

Integrins are integral membrane proteins, which mediate cell–cell, cell–extracellular matrix, and cell–pathogen adhesion. Integrin α_M_β_2,_ also known as complement receptor 3, CD11b/CD18, and macrophage-1 antigen (Mac-1), participates in all three types of interactions. The noncovalently associated subunits α_M_ (CD11b) and β_2_ (CD18) consist of a large N-terminal ectodomain, a single transmembrane helix, and a C-terminal cytoplasmic tail (1). Multiple crystal structures of the ectodomain or headpiece (HP) fragments of α_L_β_2_ (lymphocyte function–associated antigen-1, CD11a/CD18) and α_x_β_2_ (complement receptor 4, CD11c/CD18, and p150,95) form the foundation for the mechanistic understanding of β_2_-integrins (2, 3, 4). The ectodomain is divided into a HP consisting of the N-terminal domains, and the tailpiece consisting of the membrane-proximal C-terminal domains (Fig. 1*A*). α_M_β_2_ contains a von Willebrand factor type A domain, known as the αI domain, in the α-chain (5). The αI domain harbors a Mg^2+^-binding site, known as the metal ion–dependent adhesion site (MIDAS) (6, 7). The MIDAS is directly involved in ligand recognition, where a glutamate or an aspartate of the ligand coordinates the MIDAS Mg^2+^ ion (7, 8). The αI domain adopts two major conformations, open and closed (7). Transition from the closed to the open conformation leads to a rearrangement of the C-terminal α7-helix within the αI domain and a geometry of the MIDAS that permits coordination of Mg^2+^ by the ligand aspartate/glutamate (9).

**Figure 1:**
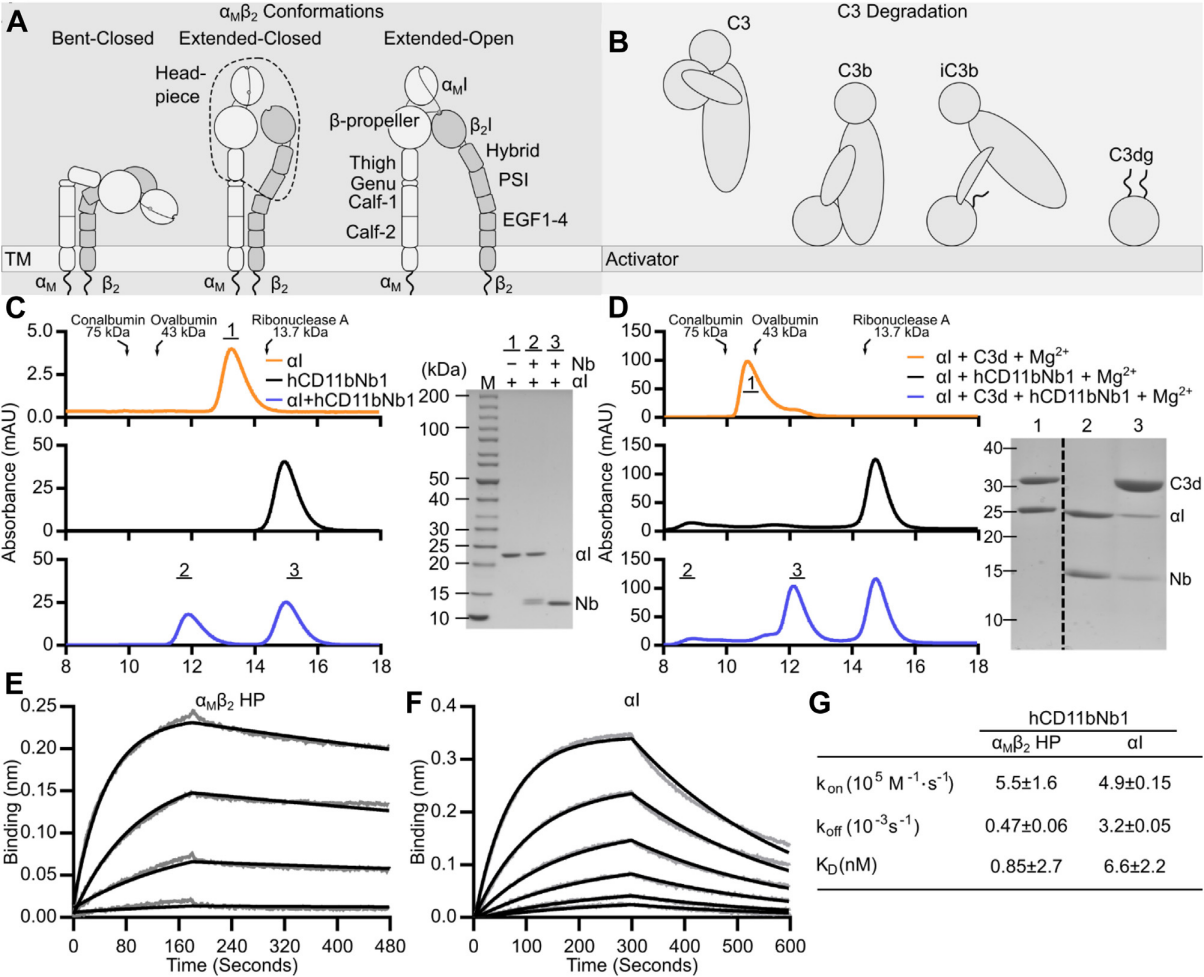
**The α**_**M**_**β**_**2**_**receptor, its iC3b ligand, and characterization of hCD11bNb1.***A* and *B*, cartoon of the three major conformations of α_M_β_2_ and the generation of iC3b upon complement activation. *C*, SEC analyses of αI (*upper panel*), hCD11bNb1 (*middle panel*), as well as both hCD11bNb1 and αI (*lower panel*). A SEC profile of the αI domain in the figure illustrates the nonideal behavior of the αI domain when not bound to a ligand in the presence of Mg^2+^; therefore, a magnesium-free buffer was employed. In the presence of the nanobody, the α_M_β_2_ αI domain elutes markedly earlier (*lower panel*). The fractions indicated by *bars* were analyzed by SDS-PAGE (*right*). The elution volumes of proteins with known molecular weight are indicated, and the void volume is at 8 ml. *D*, SEC analyses of αI with C3d (*upper panel*), hCD11bNb1 (*middle panel*), as well as both components (*lower panel*) in the presence of magnesium. SEC analysis demonstrates that the nanobody interferes with formation of the αI–C3d complex. The fractions indicated by *bars* were analyzed by SDS-PAGE (*right*). *E*, BLI analysis of immobilized hCD11bNb1 with the α_M_β_2_ HP in concentrations of 50, 25, 12.5, and 6.25 nM. *F*, as in *E*, but with the α_M_β_2_ αI domain present in concentrations of 25, 12.5, 6.25, 3.135, 1.6, and 0.8 nM. The *gray curves* in *E* and *F* are the raw data, and the *black curves* are the fitted curves. *G*, rate constants and dissociation constants calculated from BLI sensorgrams and their standard deviations derived from three independent experiments. BLI, biolayer interferometry; HP, headpiece; SEC, size-exclusion chromatography.

One primary function attributed to α_M_β_2_ is phagocytosis of complement-opsonized cells and immune complexes. Proteolytic cleavage of component 3 (C3) by convertases deposits the opsonin C3b on the activator (Fig. 1*B*). The C3b has a short half-life and is quickly converted to iC3b (10) and eventually C3dg. iC3b exhibits high affinity for α_M_β_2_, whereas the smaller C3d and C3dg fragments bind the α_M_β_2_ HP 20-fold weaker (8, 11). α_M_β_2_ is highly expressed on the plasma membrane of myeloid cells, including macrophages, monocytes, dendritic cells, and neutrophil granulocytes, and upregulated from storage granules upon stimulation. α_M_β_2_ is also highly expressed in microglia, the mononuclear phagocytes of the central nervous system, and α_M_β_2_-mediated phagocytosis of iC3b-opsonized presynaptic termini of neurons is important for neural development and homeostasis (12, 13, 14, 15). The α_M_β_2_ also plays a key role in complement stimulation of the adaptive immune system. Immune complexes containing iC3b-opsonized antigens drain into the subcapsular sinus where complement-opsonized antigens are taken up by macrophages via α_M_β_2_ and are carried across the subcapsular sinus floor (16). External stimuli, such as chemokines, cytokines, or foreign antigens, can lead to intracellular signaling, which, in turn, induces conformational changes in the integrin ectodomain to increase the ligand affinity (5) (Fig. 1*A*). In the low-affinity bent-closed state, the ectodomain conformation positions the αI close to the plasma membrane with the HP in the ligand-binding inactive conformation. In the intermediary extended-closed conformation, the integrin extends, leading to the αI domain pointing away from the plasma membrane, but the HP remains in the closed conformation. In the high-affinity extended-open state, the β_2_ hybrid domain is swung away from the α_M_ thigh domain opening the HP for ligand binding.

Within the β_2_ subunit, the βI domain is structurally homologous to the αI domain but contains two additional regulatory metal ion–binding sites (17). Structure–function studies of the α_L_β_2_ and α_X_β_2_ integrins revealed that the βI domain is responsible for relaying bidirectional signaling from or to the αI domain (18, 19). This has led to a model for the allosteric regulation of αI domain affinity. Central to this model, the Mg^2+^ in the βI domain MIDAS may become coordinated by Glu320 (mature numbering) from the α_M_ subunit located at the C-terminal region of the α7 helix of αI. Its interaction with the βI domain exerts a pull on the helix, which forces the αI domain into the open conformation during inside–out signaling. Conversely, movement of the α7 helix induced by ligand binding to the αI domain induces Glu320 coordination of the βI domain MIDAS Mg^2+^ and shifts the βI domain into the open conformation. Crystal structures indicate that the transition to the open conformation of the βI domain translates into a 60° swing out of the β_2_ hybrid domain that moves the plexin–semaphorin–integrin (PSI) domain by 70 Å (20). This swing propagates into the open-extended conformation of the β_2_ subunit, which induces intracellular signaling. These major conformational changes enable the use of allosteric antagonist of ligand to break the internal conformational signaling in β_2_ integrin ectodomains (21). It is not clear from available data if the conformational dynamics also permit functional regulation by agents affecting the steric freedom of integrins in the cell membrane environment.

Here, we present the first atomic structure of the α_M_β_2_ HP fragment in complex with the nanobody (Nb) hCD11bNb1 obtained by crystallography. The receptor adopts the closed conformation with low ligand affinity. The Nb binds to the αI domain in the α_M_ subunit in an Mg^2+^-independent manner, and biophysical experiments as well as a structural comparison suggest that it acts as a competitive inhibitor of iC3b binding. In assays with cell-bound α_M_β_2_, however, the Nb stimulates interaction with iC3b. We propose a model that integrates the entire conformational spectrum of the receptor and the dynamic properties of ligand–α_M_β_2_ complexes to reconcile these observations.

## Results

### Selection and characterization of hCD11bNb1

Nbs are single domain antibodies of typically 120 residues derived from the variable domain of heavy chain–only antibodies present in members of the Camelidae family. In addition to their potential for modulating the function of their antigen, Nbs often facilitate structure determination of challenging targets (22). Hence, we hypothesized that Nbs specific for α_M_β_2_ HP could enable its structure determination and, at the same time, lead to identification of novel modulators of the α_M_β_2_–ligand interactions. We selected Nbs against recombinant human αI domain using a phage library generated from a Llama immunized with the α_M_β_2_ HP. To favor the selection of Nbs with potential for interfering with the interaction between αI domain and C3d, we performed competitive elution with recombinant C3d. This elution strategy yielded the Nb hCD11bNb1, which we cloned into a bacterial expression vector, expressed, and purified.

We validated the interaction of hCD11bNb1 with the αI domain by size-exclusion chromatography (SEC) in the absence of Mg^2+^. The complex eluted markedly earlier than the two separate components, and SDS-PAGE analysis confirmed the presence of both components in the early peak fractions (Fig. 1*C*). Next, we investigated the effect of hCD11bNb1 on the interaction between the αI domain and C3d in the presence of Mg^2+^. After having confirmed that the αI domain and C3d form a complex stable during SEC analysis and eluting at 10.6 ml (Figs. 1*D* and S1), we tested the effect of hCD11bNb1 on the interaction. In the presence of the Nb, the peak at 10.6 ml for the αI–C3d complex disappeared. SDS-PAGE analysis confirmed that the major peak now eluting at 12 ml almost exclusively contained C3d (Fig. 1*D*).

We next measured the binding kinetics for the hCD11bNb1–α_M_β_2_ interaction using biolayer interferometry (BLI). We loaded His-tagged hCD11bNb1 on sensors coated with a His_5_-specific antibody and transferred the sensors into a solution containing either recombinant α_M_β_2_ HP or the αI domain (Fig. 1, *E*–*G*). Data analysis with a 1:1 binding model revealed that the Nb binds with a low nanomolar affinity to both the α_M_β_2_ HP and the αI domain (Fig. 1*G*). Overall, our SEC and biophysical experiments demonstrated that hCD11bNb1 binds with nanomolar affinity to the αI domain and compete with the C3d ligand, the latter a prior expectation considering the selection strategy.

### Structure determination of the α_M_β_2_ complex with hCD11bNb1

**Figure 2:**
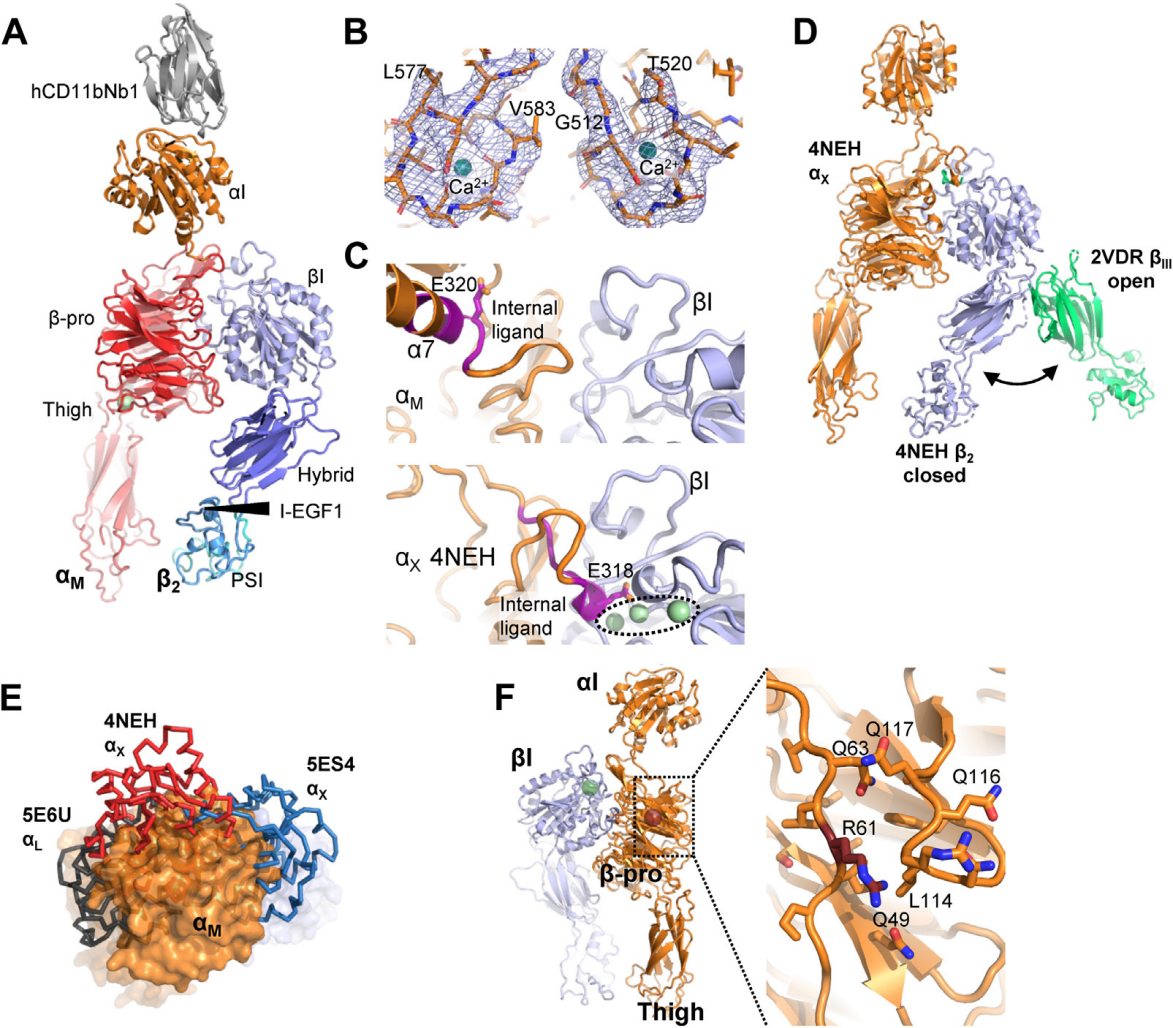
**The crystal structure of the α**_**M**_**β**_**2**_**headpiece in complex with the hCD11bNb1.***A*, *cartoon representation* of the structure with α_M_ domains in *orange* and *red*, βI domains in *blue*, and the nanobody colored *gray*. The proximity of β_2_ domains to the α_M_ thigh domains demonstrates that the structure represents the closed conformation of the headpiece. *B*, omit 2m*F*_o_–D*F*_c_ electron density around the Ca^2+^ sites in the α_M_ β-propeller contoured at 1.5 σ calculated at 3.2 Å resolution. Residues 575 to 585 and 512 to 521 from the α_M_ subunit are shown in *sticks*, and the two calcium ions were omitted for map calculation. *C*, comparison of the internal ligand region (*magenta backbone*) in our α_M_β_2_ structure and an internally liganded structure of α_X_β_2_. In the *lower panel*, three metal ions binding are *encircled*; the central ion is located in the MIDAS coordinating the internal ligand glutamate. *D*, for comparison with *A*, the closed form of α_X_β_2_ (*blue* β_2_ subunit) is displayed together with the open conformation of the headless integrin α_2_β_III_ with a *green* β-subunit. *E*, compared with known structures of β2-integrins, the αI domain (*orange*) in α_M_β_2_ is located in a unique position. *F*, *left*, overall location of Arg61 40 Å from the αI domain. To the *right*, a magnified view showing the proximity of Arg61 to the 111 to 118 loop that may adopt a different conformation and possess altered dynamic properties in the presence of a histidine at position 61. MIDAS, metal ion–dependent adhesion site.

Despite extensive screening of the α_M_β_2_ HP alone and its complex with C3d or iC3b, we failed to obtain useful crystals or nonaggregated particles on cryo-EM grids; in the latter case, most likely because of the effects of the air–water interface (23). However, when bound to the Nb, the α_M_β_2_ HP readily crystallized in a number of different organic salts capable of chelating Mg^2+^ ions. We obtained seven X-ray diffraction datasets with synchrotron radiation that extended to a maximum resolution of 3.2 Å, but all these suffered from strong anisotropy. From all these datasets, we were able to determine the structure by molecular replacement using the coordinates of the β-propeller of α_X_β_2_ or the βI domain of α_L_β_2_ as search models. The resulting electron density and comparison with the structures of α_X_β_2_ and α_L_β_2_ enabled us to place the α_M_ thigh domain and the β_2_ domains hybrid, PSI, and integrin epidermal growth factor 1 (I-EGF1). We also identified electron density that could be manually fitted with a model of the Nb bound to its epitope in the αI domain. The electron density calculated from the resulting model and noncorrected diffraction data was of low quality in the receptor proximal part of the thigh domain, the PSI and I-EGF1, and at C-terminal pole of the Nb irrespective of the used dataset. Data were therefore scaled anisotropically with the STARANISO server (Global Phasing Ltd) (24), which led to a significant improvement of both 2m*F*_o_–*F*_c_ and density-modified electron density maps. The dataset exhibiting the best statistics after refinement of an initial model was selected for completion of the structure. To support the modeling of the α_M_β_2_–Nb complex, we also determined the structure of hCD11bNb1 itself based on diffraction data extending to a resolution of 1.14 Å (Table 1 and Fig. S2, *A*–*C*). Using the anisotropy-corrected data, we refined the complex structure to an *R*_free_ value of 0.295 (Table 1). Figure 2, *A* and *B* displays the resulting structure and an example of the electron density presenting the Ca^2+^ sites in the α_M_ β-propeller. As judged from comparison with known structures of α_X_β_2_ and α_L_β_2_ together with our high-resolution structure of the Nb, large errors are unlikely in the model. The slightly elevated *R*_free_ value is probably caused by data anisotropy. The refined temperature factors are comparatively high for the C-terminal end of the thigh domain, the C-terminal end of hCD11bNb1, and the PSI/EGF1 domains as compared with the rest of the structure, most likely because of a lack of crystal packing in those areas.

**Table 1:** Data collection and refinement statistics

Structure	α_M_β_2_–hCD11bNb1 (PDB ID: 7P2D)		hCD11bNb1 (PDB ID: 7NP9)
Wavelength	0.9762		0.9763
Resolution range	44.63–3.20 (3.35–3.20)	44.63–3.5 (3.625–3.5)	30.81–1.14 (1.18–1.14)
Space group	P3_1_21		I4_1_22
Unit cell	114.1 114.1 250.12 90 90 120		82.78 82.78 72.46 90 90 90
Total reflections	1136,124 (151,037)	968,232 (139,390)	1,995,838 (90,712)
Unique reflections	31,887 (4057)	24,591 (3390)	44,396 (3194)
Multiplicity	35.6 (37.2)	35.2 (36.6)	45.0 (27.6)
Completeness (%)	99.9 (99.9)	99.9 (99.9)	96.37 (70.55)
Mean I/sigma(I)	20.69 (1.26)	26.53 (3.74)	21.50 (1.82)
Wilson *B*-factor	128.3	129.8	12.24
*R*_merge_	0.114 (3.48)	0.096 (1.33)	0.1591 (2.63)
*R*_meas_	0.116 (3.53)	0.097 (1.34)	0.1609 (2.68)
CC1/2	1 (0.865)	1 (0.98)	1 (0.484)
Reflections refinement	26,420 (1303)	23,536 (1559)	44,286 (3194)
Reflections *R*_free_	1130 (55)	1011 (70)	2006 (145)
*R*_work_	0.2605 (0.3466)	0.2479 (0.3253)	0.1462 (0.2322)
*R*_free_	0.2950 (0.4033)	0.2888 (0.3335)	0.1669 (0.2641)
CC (work)	0.911 (0.583)	0.911 (0.603)	0.974 (0.809)
CC (free)	0.851 (0.427)	0.858 (0.651)	0.958 (0.672)
Nonhydrogen atoms	10,419		1175
RMS (bonds)	0.006	0.005	0.010
RMS (angles)	1.16	1.03	1.16
Ramachandran plot			
Favored (%)	95.62	95.77	99.17
Allowed (%)	4.23	4.15	0.83
Outliers (%)	0.15	0.08	0.00
Rotamer outliers (%)	2.04	1.77	0.00
Clashscore	4.56	5.09	3.48
Average *B*-factor	127.62	136.55	18.04

Statistics for the highest-resolution shell are shown in parentheses. For the αMβ2–hCD11bNb1 complex, the data collection statistics were calculated by XSCALE from the diffraction data not corrected for anisotropy. Refinement statistics were calculated from the data corrected for anisotropy by the STARANISO server. The deposited structure in PDB entry 7P2D was refined to a maximum resolution of 3.2 Å; statistics for a maximum resolution of 3.5 Å resolution is presented for comparison.

### Overall structure of the α_M_β_2_–hCD11bNb1 complex

**Figure 3:**
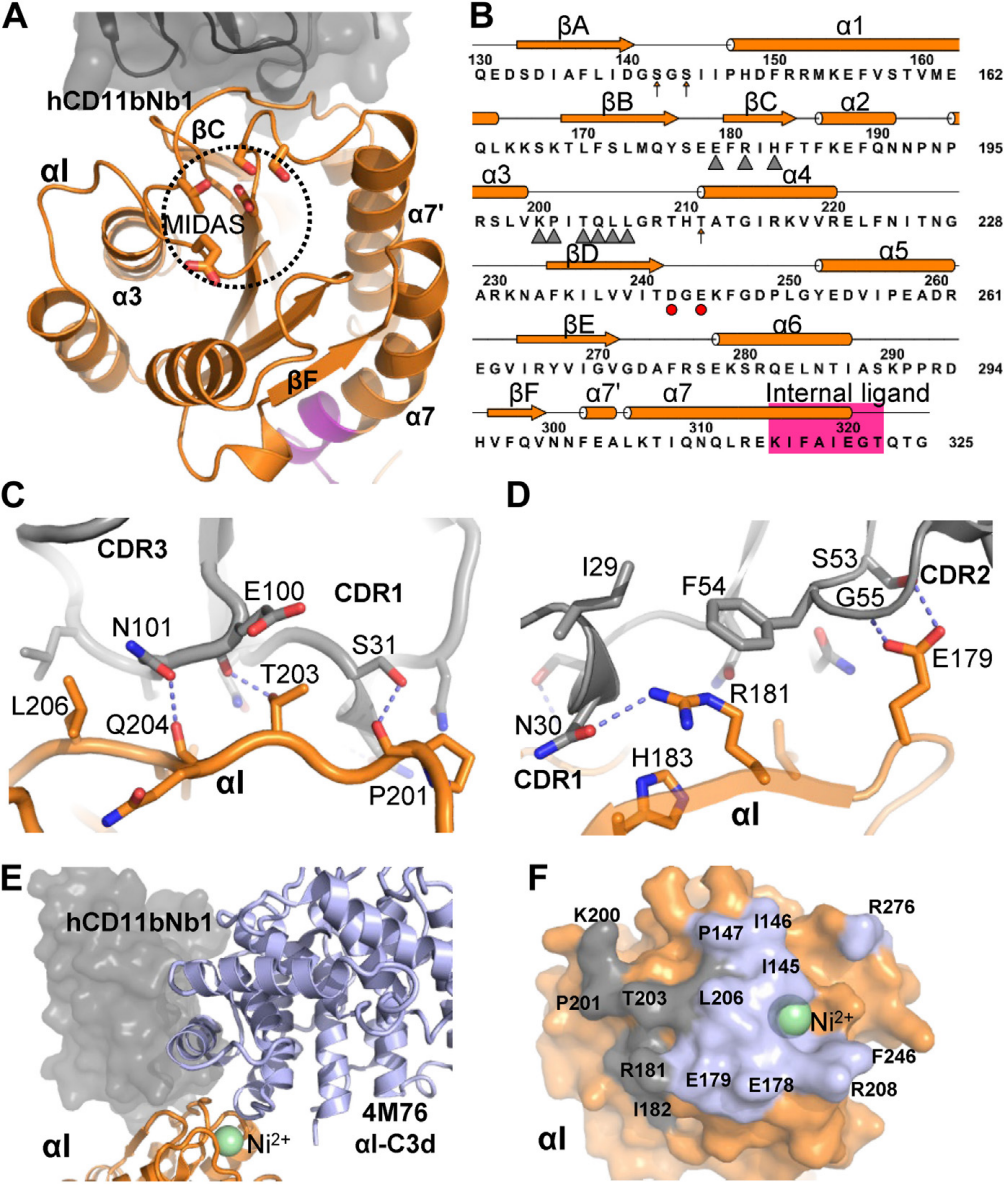
**The hCD11bNb1 epitope is located adjacent to the αI MIDAS.***A*, top view of α_M_β_2_ αI domain with the nanobody (Nb) bound with the empty MIDAS outlined. Notice also the opposite location of the Nb relative to α-helix α7 undergoing large conformational changes during transitions between the closed and open conformations. *B*, secondary structure and sequence of the αI domain, epitope residues are marked by *triangles*. MIDAS residues are outlined with *arrows* and *red circles*. *C* and *D*, details of the interaction between the αI and the Nb. *Dashed line* indicates putative hydrogen bonds. *E*, comparison of the hCD11bNb complex with our prior structure of the core complex αI–C3d created by superposition of the αI domains (*orange*). A small but significant overlap between C3d (*blue cartoon*) and hCD11bNb1 (*gray surface*) is predicted for a ternary complex. *F*, the footprint of C3d (*blue*) on the αI domain (PDB entry: 4M76) is continuous with the Nb footprint (*gray*) on the αI domain. Notice that to increase complex stability, a Ni^2+^ ion was present in the MIDAS of the αI–C3d complex structure (8) instead of a Mg^2+^ ion. MIDAS, metal ion–dependent adhesion site; PDB, Protein Data Bank.

The two subunits associate through an extensive intermolecular interface formed between the β-propeller in α_M_ and the βI domain in the β_2_ subunit, with a buried surface area of 3650 Å^2^. Superposition of the α_M_β_2_ onto structures of α_L_β_2_ and α_X_β_2_ revealed that the β-propeller and the βI domains interact in an almost identical manner across the three β_2_ receptors (Fig. S3*A*). The orientation of the thigh domain relative to the β-propeller is also quite similar in α_x_β_2_ and α_M_β_2_ (Fig. S3*B*). In the α_M_ β-propeller, two calcium ions organize coordinating loop regions that together with the first residue of α_M_ forms the interface with the thigh domain (Fig. 2*B*). In contrast, the metal ion–binding sites in both the αI and βI domains are empty, and the internal ligand region at the C-terminal end of the αI α7 helix is not interacting with the βI MIDAS site (Fig. 2*C*). This is an important notion, as ligand binding to the αI domain induces movement of the α7 helix. In turn, α_M_ Glu320 may coordinate the metal ion within the βI MIDAS site and induce the ligand-bound open conformation of the βI domain that propagates into the extended-open conformation of α_M_β_2_ (Fig. 1*A*). For this reason, the region around Glu320 is known as the internal ligand (Fig. 3*B*). The overall conformation of the α_M_β_2_ HP is closed with the hybrid, PSI, and I-EGF1 domain in the β_2_ subunit located toward the α_M_ thigh domain, in contrast to the open conformation of the β_2_ subunit known from a structure of ligand-bound α_II_β_3_ integrin (Fig. 2*D*). Within the β_2_ subunit, the arrangement of the four domains in the closed conformation of α_M_β_2_ is also highly similar to those observed for α_X_β_2_ and α_L_β_2_ (Fig. S3*C*).

### Structural basis for the altered allosteric coupling in the α_M_ R77H variant

A single nucleotide polymorphism resulting in the substitution of Arg77 to histidine in the α_M_ subunit β-propeller predisposes the carrier for systemic lupus erythematosus (SLE) (25). The R77H mutation neither does change the surface expression of α_M_β_2_ on neutrophils and monocytes nor does effect the inside–out signaling (26, 27). However, the mutation interferes with outside–in signaling since it significantly decrease phagocytosis of iC3b-opsonized red blood cells by macrophages and cell lines expressing α_M_β_2_ (26, 27). In addition, the monocytes carrying the mutated α_M_β_2_ adhere less efficiently to surfaces coated with iC3b, fibrinogen, intercellular adhesion molecule 1 (ICAM-1), ICAM-2, and DC-SIGN (dendritic cell–specific intercellular adhesion molecule-3-grabbing nonintegrin). Furthermore, R77H monocytes stimulated with a Toll-like receptor 7/8 agonist exhibit a significantly smaller decrease in cytokine secretion upon binding of iC3b-opsonized red blood cells compared with WT monocytes (26). Biomembrane force probe experiments revealed that the α_M_ R77H variant of the α_M_β_2_ receptor fails to respond to force with formation of catch bonds normally be induced when cells adhering through α_M_β_2_ to an immobilized ligand are exposed to an external force (28).

In our structure, Arg61 (the mature numbering of Arg77 after release of the propeptide) is exposed on the edge of the α_M_ β-propeller (Fig. 2*F*). The side chain of Arg61 only appears to interact with the nearby loop Gly111–Pro118 by nonspecific van der Waals interactions. The arginine side chain does not engage in specific hydrogen bonds or electrostatic interactions that could directly explain the altered allosteric coupling in α_M_β_2_ containing the R77H variant. In our crystal structure, Arg61 is located ∼40 Å from both the α7 helix in the αI domain and the MIDAS site in the βI domain. Hence, a direct interaction of Arg61 with residues in the α_M_–β_2_ interface involved in the allosteric coupling between ligand binding and transition to the extended-open conformation cannot explain the observed functional defects. Furthermore, based on the structure of the closed-bent conformation α_X_β_2_ (2, 3), we also predict that Arg61 does not interact with other domains in either of the two subunits in the bent-closed conformation of α_M_β_2_.

However, a possible consequence of a histidine at position 61 is that the neighboring 111 to 118 loop (Fig. 2*F*) changes conformation and dynamic properties because of perturbation of van der Waal interactions formed by Arg61. Alternatively, a histidine side chain at position 61 may engage in hydrogen bonds with the 111 to 118 loop. In support of an altered conformation of the 111 to 118 loop, we notice that in α_X_ with a glycine residue corresponding to α_M_ Arg61, the equivalent loop adopts a different conformation and is not in contact with the region containing the glycine. An altered conformation of the 111 to 118 loop could propagate and influence the dynamic properties of the N-terminal linkage between the β-propeller domain and the αI domain in residues 123 to 129. Alternatively, such conformational changes could propagate to α_M_ residues located at the interface to the βI domain such as the loop region Thr96–Thr101. Transmission of force is crucially dependent on a stable α_M_–β_2_ interface; even a small perturbation may give rise to the observed abnormal outside–in signaling in the R77H α_M_ variant.

### The αI domain helix 7 adopts the closed conformation

Prior structures of α_X_β_2_ and α_L_β_2_ revealed that the α-subunit β-propeller and the βI domain form a platform above which the αI domain has considerable freedom to orientate in response to crystal packing and the βI coordination state of the α_M_ internal ligand region (2, 3, 4). Confirming this idea, and in contrast to the highly conserved arrangement of the remaining domains discussed previously, the orientation of the αI domain in our structure of α_M_β_2_ is unique. Compared with structures of α_X_β_2_ with the α_x_ internal ligand region interacting with the βI MIDAS, the αI domain is rotated by 180° (Fig. 2*E*). When comparing our α_M_β_2_ structure to α_X_β_2_ and α_L_β_2_, where the internal ligand region is not contacting the β_2_ MIDAS, the αI domain is rotated by 125° and 42°, respectively (Fig. 2*E*). The α_M_β_2_-specific orientation of the αI domain may well be a result of crystal packing, since the αI-hCD11bNb1 part of the complex firmly contacts three symmetry-related complexes (Fig. S3*D*). Supporting this, there are no specific interaction between the N-terminal linker region (α_M_ residues 123–131) and the platform. At the C-terminal linker region (residues 321–328), only a putative hydrogen bond between Thr322 and a sugar residue from the glycan attached to Asn375 appears to be specific for the α_M_β_2_ structure.

The αI domain has no clear density for a Mg^2+^ ion although it was available during crystallization. Also, the electron density suggests that the main-chain conformation of residues Asp242–Glu244 may not be fixed. As this region differ between the open and closed forms of the αI domain (7), the conformation of the MIDAS site itself cannot be defined (Fig. 3*A*). Hence, the Nb does not appear to depend on a particular MIDAS conformation, and SEC analysis confirms that its binding to the αI domain is Mg^2+^ independent (Fig. 1, *C* and *D*). This is also consistent with that the conformation of the epitope described later does not differ significantly in structures representing the open and closed states of the αI domain.

Another signature of the αI domain conformational state is the length and position of the α7 helix (7). In the Nb complex, the α_M_ Phe302–Glu320 region is in a helical conformation (Figs. 3, *A* and *B*, S3*E*) meaning that this region adopts the closed conformation that prevents Glu320 from interacting with the βI MIDAS. Since hCD11bNb1 and the α7 helix are located oppositely on the αI domain (Fig. 3*A*) and the Nb apparently does not induce a specific conformation of the αI MIDAS, the Nb is unlikely to influence the conformation of the α7 helix significantly. Its closed conformation is more likely to be a result of the crystal packing that favors the overall closed conformation of the β_2_ subunit in the βI domain incompatible with binding of the α_M_ internal ligand region. In summary, both the αI domain α7 helix and the overall conformation of the α_M_β_2_ HP signify the closed conformation; however, this appears not to be a consequence of the Nb. In solution, the α_M_β_2_ HP contains a mixture of the open and closed conformations (11) that are likely to bind the Nb with very similar affinities.

### The Nb epitope on the αI domain is proximal to the C3d-binding site

The quality of the electron density for the Nb–αI interface is overall good considering the resolution and data anisotropy (Fig. S3*D*). Furthermore, known structures of the αI domain and our own 1.14 Å-resolution structure of hCD11bNb1 itself (Fig. S2*A*) considerably facilitated modeling of the intermolecular interface. The buried surface area of the interface is 1330 Å^2^, which is low, but not unusual, when compared with most other Nb–antigen complexes (29). The interface is dominated by polar interactions (Fig. 3, *C* and *D*). Extensive burial of hydrophobic side chains at the Nb–antigen interface plays a prominent role in our complexes of Nbs with complement C3b, C4b, and C1q (29, 30, 31) but is not observed in this case, although the hCD11bNb1 Phe54 stacks with the guanidinium group of αI Arg181 (Fig. 3*D*). The epitope of the Nb comprises two distinct regions in the αI domain. First, Nb complementarity-determining regions (CDRs) 1 and 3 recognize αI residues Pro201–Leu206 with hydrogen bonds and van der Waals interactions (Figs. 3*C* and S2*D*). Second, αI residues in the region Glu179–His183 encompassing β-strand C interact with hCD11bNb1 CDR1 and CDR2 through van der Waals interactions and hydrogen bonds (Figs. 3*D* and S2*D*).

Interestingly, a comparison of the αI–CD11bNb1 and the αI–C3d (8) complexes revealed a small, but significant, overlap between the Nb and C3d suggesting that their binding is mutually exclusive (Fig. 3, *E* and *F*). Specifically, the Nb CDR3 residues 103 to 107 are predicted to exert steric hindrance on C3d residues Ala1214–Lys1217 in a loop region at the end of a C3d α-helix. Since the thioester domain of iC3b is expected to bind α_M_β_2_ in the same manner (8, 11), this predicts that the Nb interferes with both α_M_β_2_–iC3b and α_M_β_2_–C3d interactions. In summary, our structural analysis defined the paratope and epitope and their interactions in details and predicted that hCD11bNb1 acts as a competitive inhibitor of iC3b and C3d binding to α_M_β_2_ by exerting steric hindrance on the thioester domain of the ligand.

### Biophysical analysis of the iC3b–α_M_β_2_ complex confirms the crystal structure

**Figure 4:**
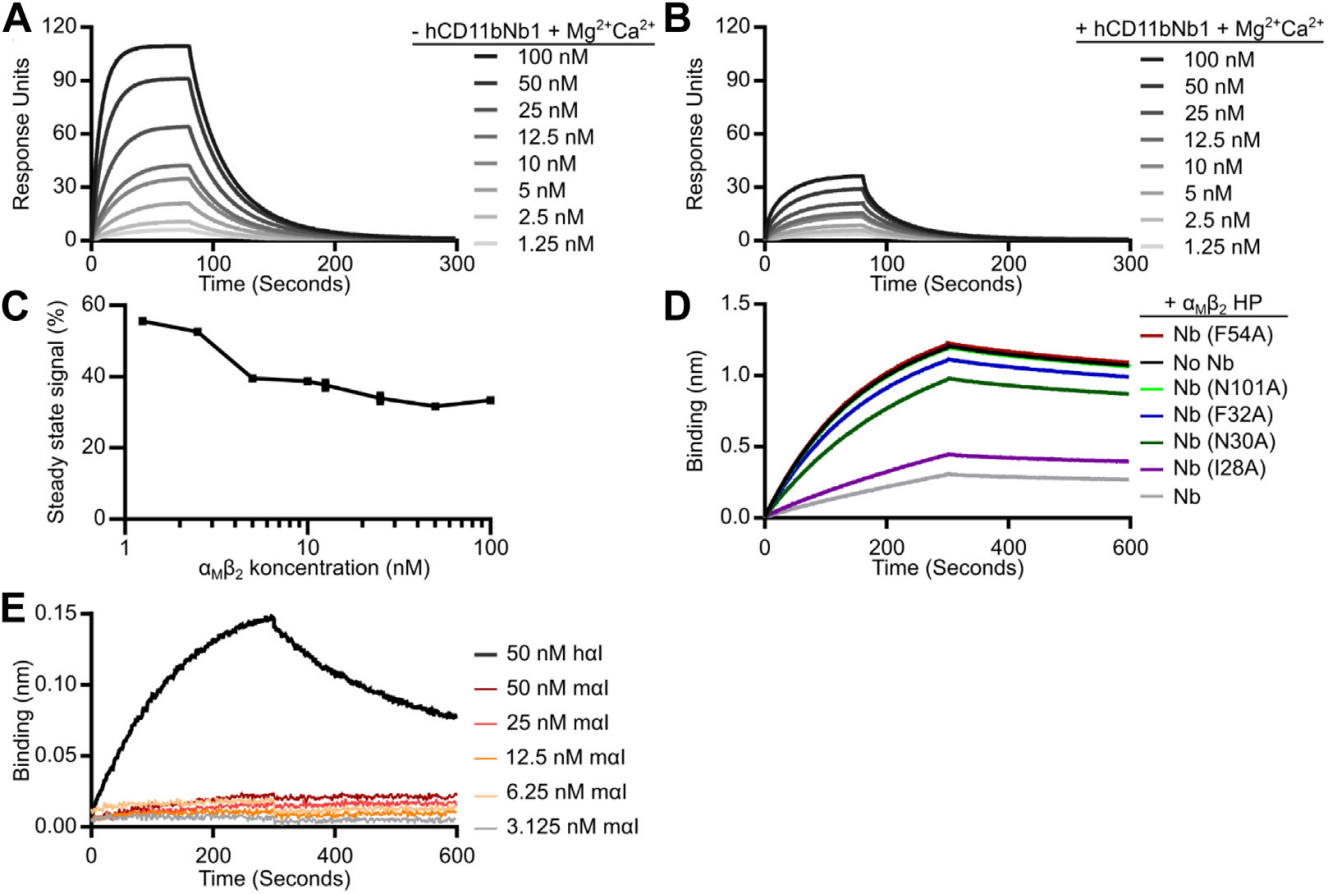
**Characterization of the effect of hCD11bNb1 on the α**_**M**_**β**_**2**_**–iC3b interaction.***A* and *B*, biotinylated iC3b was immobilized on a streptavidin-loaded SPR chip. Next, α_M_β_2_ HP in the indicated concentrations in either presence (+hCD11bNb1) or absence (−hCD11bNb1) of a 1.5-fold molar excess of hCD11bNb1 was injected. *C*, quantitation of the decrease in α_M_β_2_ HP binding to iC3b in *B* with hCD11bNb1 present compared with *A* without hCD11bNb1 present. *D*, the effect of mutations in nanobody CDRs. Biotinylated avi-tagged hCD11bNb1 was immobilized on streptavidin biosensors, and the sensors were transferred into 20 nM α_M_β_2_ HP alone or 20 nM α_M_β_2_ HP preincubated with 10-fold molar excess of hCD11bNb1 mutants I29A, N30A, F32A, F54A, N101A, or WT hCD11bNb1. *E*, BLI-based analysis of the interaction between hCD11bNb1 and either human or murine αI domain. hCD11bNb1 was immobilized on anti–penta-HIS sensors, and the sensors were transferred into human αI domain (hαI) at 50 nM or the murine αI domain (mαI) at 50, 25, 12.5, 6.25, or 3.125 nM. BLI, biolayer interferometry; CDR, complementarity-determining region; HP, headpiece; SPR, surface plasmon resonance.

To test the prediction that hCD11bNb1 acts as a competitive inhibitor for the α_M_β_2_–iC3b interaction, we took advantage of the high-affinity monovalent interaction occurring between iC3b and the α_M_β_2_ HP (11). We biotinylated the free cysteine appearing in nascent C3b upon thioester cleavage using a maleimide–biotin reagent, converted the C3b to iC3b, and coupled the biotinylated iC3b to a streptavidin-loaded surface plasmon resonance (SPR) sensor. This strategy presents iC3b in the geometry that it would have on an activator after C3b deposition and factor I degradation as outlined in Figure 1*B*. We next flowed recombinant α_M_β_2_ HP over the iC3b-coated sensor in the presence or the absence of a 1.5-fold molar excess of hCD11bNb1. As previously reported (11), we observed a *K*_*D*_ value of 30 nM in the absence of the Nb (Fig. 4*A*). In the presence of hCD11bNb1, the signal decreased to 32 to 55% of the signal obtained in the absence of the Nb (Fig. 4, *B* and *C*), demonstrating that the Nb acts as an inhibitor of iC3b for binding to the α_M_β_2_ HP.

To validate the interface between hCD11bNb1 and the α_M_β_2_ HP observed in the crystal structure, we mutated Nb residues in direct contact with the receptor (Asn30, Phe54, and Asn101) or in the vicinity (Ile29 and Phe32) likely to support the conformation of the directly interacting residues. We next coupled biotinylated WT hCD11bNb1 to streptavidin-coated BLI sensors and carried out a competition assay where variants of hCD11bNb1 were present in the fluid phase in 10-fold molar excess to the α_M_β_2_ HP. As expected, the presence of the parental hCD11bNb1 in the fluid phase reduced the binding to approximately 22% of the signal obtained with the α_M_β_2_ HP only (Fig. 4*D*). The Ile29Ala variant competed almost as well as the parental Nb, whereas the remaining variants more or less lost the ability to inhibit binding of the α_M_β_2_ HP to the immobilized hCD11bNb1 (Fig. 4*D*). Overall, our experiments with mutated Nb variants validated the paratope–epitope interaction deduced from the crystal structure.

Since hCD11bNb1 appeared to modulate the function of α_M_β_2_, we investigated whether the Nb could bind to the recombinant αI domain from the murine α_M_ subunit. If hCD11bNb1 also modulates the activity of murine α_M_β_2_, it may be an attractive reagent for *in vivo* murine models of pathogenesis where the receptor plays a role as discussed later. We charged anti-His BLI sensors with His-tagged hCD11bNb1 and compared the binding of the recombinant murine αI domain to the human αI domain. While we observed a strong signal for the human domain, the murine αI domain bound much weaker (Fig. 4*E*), and the data could not be fitted to obtain rate constants or the *K*_*D*_ value. To understand why the Nb binds the murine αI domain much weaker, we constructed a homology model of the murine domain. Inspection of this model suggested two reasons for our observations. First, a lysine in mouse αI substitutes for Thr203 in the human αI domain. This is likely to lead to steric hindrance when the Nb binds to the murine domain. Second, L206 in the human αI domain making contact with CDR3 of the Nb is replaced by the polar N222 in murine α_M_, which may also decrease affinity. In conclusion, our biophysical experiments agreed with the predictions made from the crystal structure of the complex and demonstrated that hCD11bNb1 is unlikely to modulate the activity of murine α_M_β_2_.

**Figure 5:**
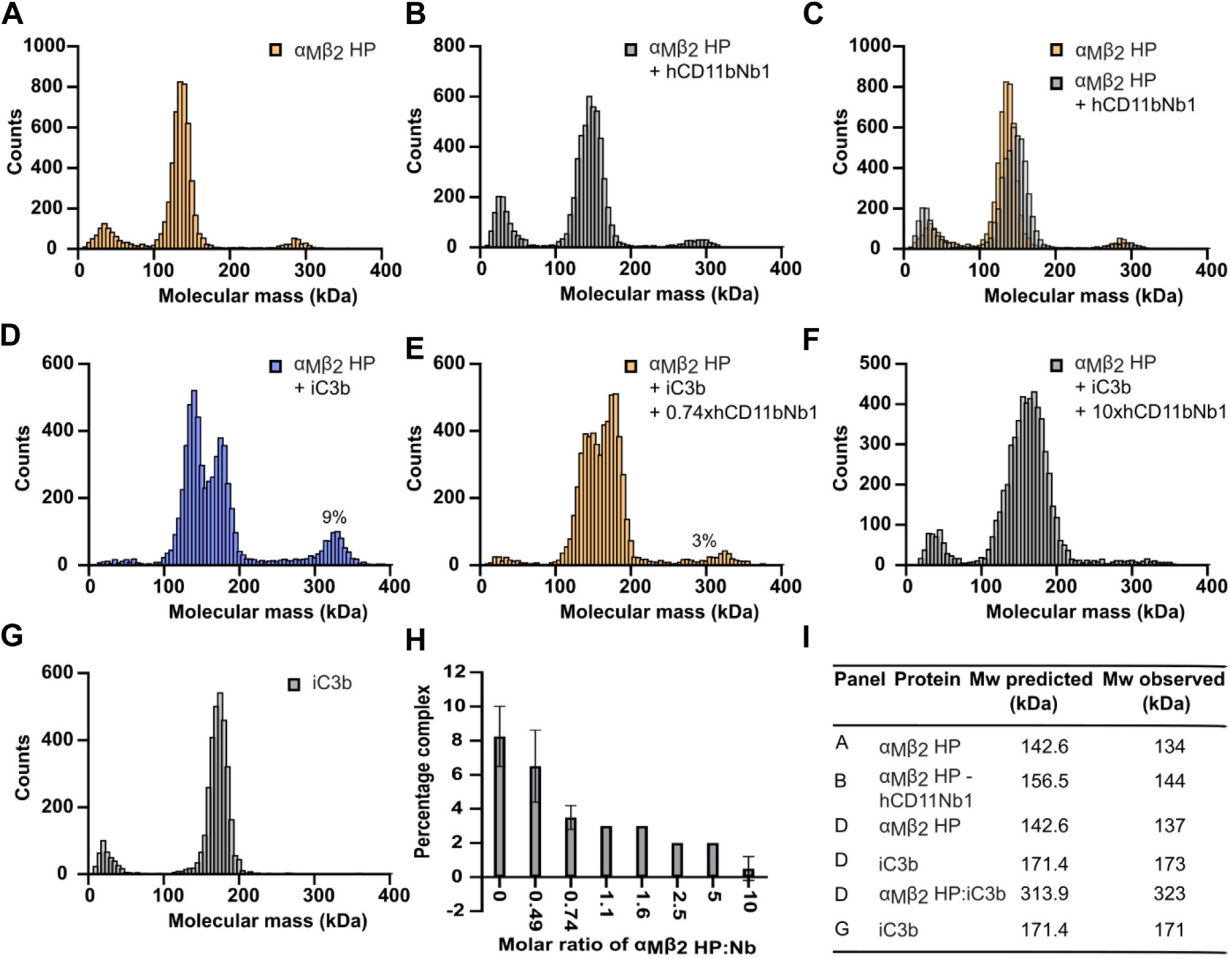
**Characterization of α**_**M**_**β**_**2**_**HP, iC3b, and the influence of hCD11bNb1 in solution measured by mass photometry.***A*, mass distribution of 10 nM α_M_β_2_ HP. The signal below 50 kDa is due to noise and present in all measured curves. The small peak at 285 kDa is likely to correspond to a small amount of α_M_β_2_ HP dimers as described (11). *B*, 10 nM α_M_β_2_ HP with 100 nM hCD11bNb1. The nanobody does not induce oligomerization of the α_M_β_2_ HP. *C*, overlay of the two MP distributions in *A* and *B*. *D*, 10 nM α_M_β_2_ HP mixed with 10 nM iC3b gives rise to a peak for complex at 323 kDa. The percentage of complex formed corresponds to the area of the complex peak compared with the total area under the curve including the area under the curve below 100 kDa as calculated by the DiscoverMP software. *E*, as in *D* with 7.4 nM hCD11bN1. Partial dissociation of the complex is evident. *F*, as in *D* with 100 nM hCD11bN1. The peak for the complex is now insignificant. *G*, mass distribution of 10 nM iC3b. *H*, bar chart displaying the percentage of α_M_β_2_ HP–iC3b complex as a function of the molar ratio between α_M_β_2_ HP and the nanobody at a fixed 10 nM concentration of α_M_β_2_ HP and iC3b. The ratios are based on the average of two experiments. For some ratios, the percent complex reported as an integer number was identical. *I*, table of molecular masses observed that extracted from the mass distributions. HP, headpiece.

To obtain more insight in the mechanism of inhibition, we investigated whether hCD11bNb1 induced aggregation of the α_M_β_2_ HP leading to release of iC3b or acted as a partial antagonist in a mechanism distinct from the competitive inhibition suggested by our structural analysis. Since neither BLI nor SPR reveal the oligomerization state of the fluid phase analyte, we took advantage of mass photometry (32) to study how increasing concentrations of hCD11bNb1 affects the α_M_β_2_ HP–iC3b complex. The mass photometry contrast-count curves recorded at a concentration of 10 nM for iC3b and α_M_β_2_ HP revealed molecular weights in good agreement with their predicted values (Fig. 5). As expected, the receptor and iC3b formed a monomeric 1:1 complex in agreement with our recent SEC and small-angle X-ray scattering analysis of the complex (11). In the absence of iC3b, the α_M_β_2_ HP and the Nb formed a monomeric complex demonstrating that hCD11bNb1 does not induce aggregation of the α_M_β_2_ HP (Fig. 5, *B* and *C*). Titration of the α_M_β_2_ HP–iC3b complex with increasing amounts of hCD11bNb1 leads to dissociation of the complex (Fig. 5, *D*–*F*). At 10-fold molar excess, complex dissociation appeared to be complete (Fig. 5*F*). Overall, our mass photometry data supported that hCD11bNb1 acts as a competitive inhibitor of iC3b binding to the α_M_β_2_ HP.

### hCD11bNb1 stimulates the interaction of α_M_β_2_ presenting cells with iC3b

**Figure 6:**
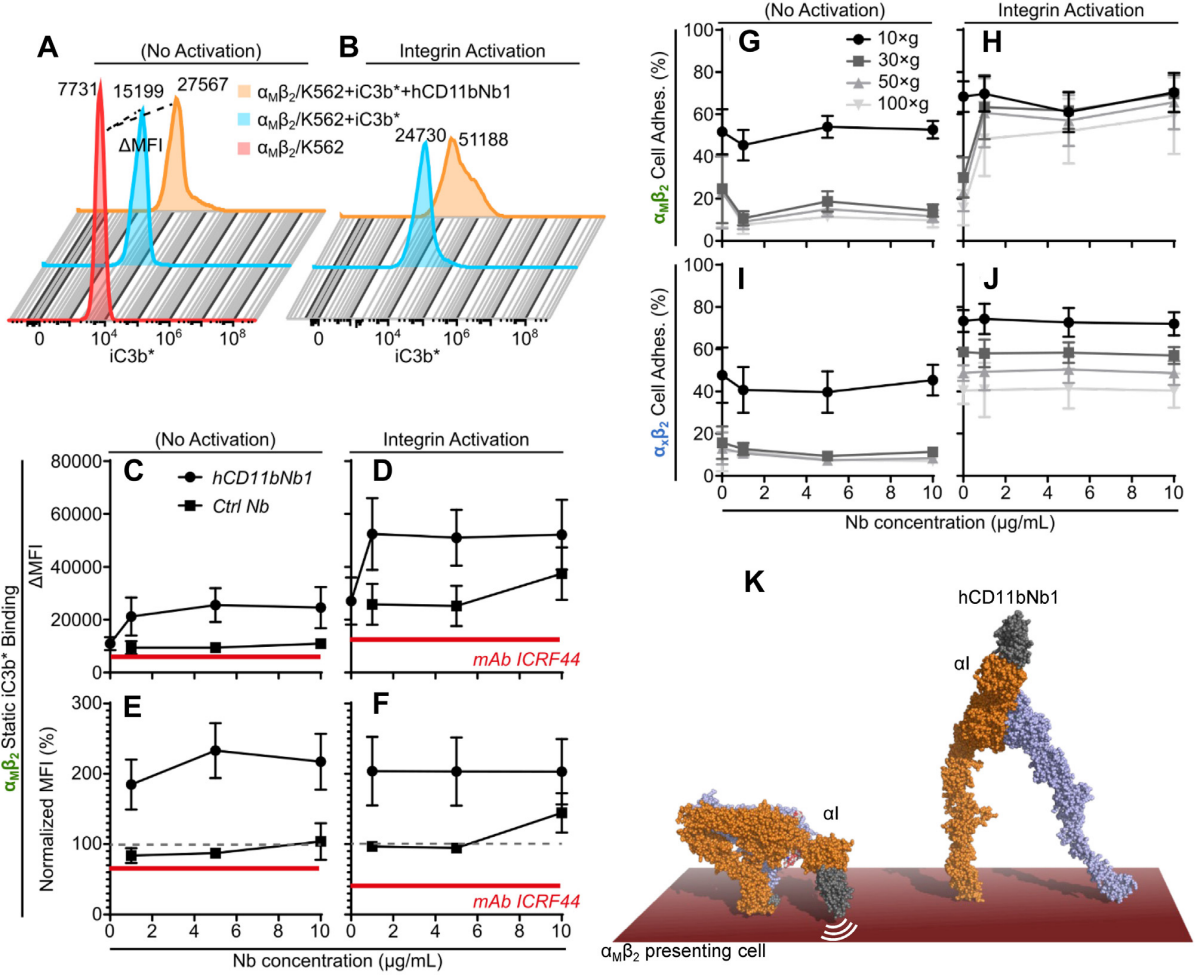
**Binding of iC3b to cell-expressed αMβ2 is stimulated by hCD11bNb1.** The influence of hCD11bNb1 on K562 cell–expressed recombinant α_M_β_2_ ligand binding was tested in a static assay permitting equilibrium to be reached (*A–F*) or a force-based cell adhesion assay (*G*–*J*), in both cases with iC3b as the ligand. *A*–*F*, static binding of fluorescent-labeled iC3b (iC3b∗) to α_M_β_2_. *A* and *B*, raw data for a representative experiment with an indication of the calculation of ΔMFI by subtraction of the autofluorescence MFI (“α_M_β_2_/K562”) from the MFI for cells with iC3b∗ (“α_M_β_2_/K562 + iC3b∗”) or iC3b∗ and hCD11bNb1 (“α_M_β_2_/K562 + iC3b∗ + hCD11bNb1”) under integrin resting (*A*) or ligand-binding activating conditions (*B*). *C* and *D*, the ΔMFI was calculated for cells mixed with iC3b∗ and 0, 1, 5, or 10 μg/ml of hCD11nNb1, either under integrin resting (*C*) or activating conditions (*D*). As a control, nonbinding nanobody (Nb) was applied in the same amounts. *E* and *F*, the data were also shown as normalized to the iC3b∗ without Nb addition (≡100%, indicated with a *gray hatched line*). *C*–*F*, the influence of the function blocking monoclonal antibody (mAb) ICRF44 to the α_M_ chain is indicated with *red solid lines*. *G*–*J*, influence of hCD11bNb1 on iC3b binding in a force-based cell adhesion assay. α_M_β_2_/K562 cells were applied V-shaped wells coated with iC3b together with 0, 1, 5, or 10 μg/ml of hCD11bNb1. Increasing centrifugational force was applied in sequential steps of 10, 30, 50, and 100*g* (indicated with *gray* coloring of curves). As a control, the influence of hCD11bNb1 on the binding by α_X_β_2_/K562 to iC3b was also tested as for the experiments with α_M_β_2_/K562. For each experiment in *C*–*J*, the adhesion was calculated as the mean of triplicates. The results shown are the mean and SEM for three independent experiments (N = 3). *K*, models of α_M_β_2_ on a cell membrane. The hCD11bNb1 possibly interferes with the bent-closed conformation (*left*) because of the proximity of αI domain and the associated Nb to the cell membrane. This may shift the conformational equilibrium of the proteins embedded in the membrane toward the extended conformation with high ligand affinity (*right*). MFI, mean fluorescence intensity.

We next asked whether the competition between the Nb and iC3b observed in binding experiments with isolated components and rationalized by structural comparison could be translated to the full α_M_β_2_ receptor on cells. The binding of the iC3b ligand to α_M_β_2_ was evaluated in two types of cell-based assays. The first assay was based on the binding of fluorescent-labeled iC3b (iC3b∗) incubated with K562 cells that express α_M_β_2_. Here, the long incubation time permitted the reaction to reach equilibrium (11), and the fluorescent signal from cell-bound iC3b∗ was quantitated by flow cytometry. The signal obtained for incubations with iC3b∗ alone or together with hCD11bNb1 was subtracted. Autofluorescence (Δ mean fluorescence intensity) was measured in cells with no addition of iC3b∗, either for conditions without integrin activation (Fig. 6*A*) or with Mn^2+^ added to activate ligand binding (Fig. 6*B*). In this assay, the influence of hCD11bNb1 showed a dose-dependent increase of ∗iC3b both under conditions with and without integrin activation (Fig. 6, *C* and *D*). Importantly, an unrelated control Nb did not increase the binding quantitatively. When this binding was normalized to the signal with no hCD11bNb1 (Fig. 6, *E* and *F*), it became clear that the observed increase in iC3b∗ binding was independent of integrin activation: in both cases, the stimulation was approximately twofold higher in the presence of hCD11bNb1. Especially for nonactivating conditions (Fig. 6*E*), the binding signal followed the hCD11bNb1 concentration with lower signal for a concentration of 1 μg/ml (∼75 nM) compared with 5 or 10 μg/ml, which showed signs of saturation.

Our second cell-based assay involved force exerted through centrifugation of V-shaped microtiter wells coated with iC3b. In this way, adherent cells transmit a force load onto the ligand-bound α_M_β_2_ mimicking physiological conditions, where sheer stress acts similarly (33). Adhesion of K562 cell–expressing α_M_β_2_ to the iC3b-coated wells increased with addition of hCD11bNb1; however, with no sign of titration of the signal in the hCD11bNb1 concentration range was investigated. Interestingly, in this case, the increase only occurred under integrin-activating conditions in an applied force regiment from 30 to 100*g* (Fig. 6, *G* and *H*). As a control, we tested α_X_β_2_-expressing K562 cells, which also bind iC3b in the centrifugation assay. In this case, the hCD11bNb1 had no influence on the cell adhesion, consistent with the absence of the α_M_ chain (Fig. 6, *I* and *J*). Overall, the cell-based assays demonstrated that hCD11bNb1, through its binding to the αI domain, can both stimulate the interaction of α_M_β_2_ with fluid phase monovalent iC3b as well as the multivalent interaction between α_M_β_2_-presenting cells and an iC3b-coated surface. Hence, with the receptor presented on the cell surface, hCD11bNb1 acted as new protein-based α_M_β_2_-specific agonist that promotes interaction with iC3b.

### A model for hCD11bNb1 stimulation of iC3b binding

As noted previously, the hCD11bNb1 showed an inhibitory effect on ligand binding to the α_M_β_2_ HP and the isolated αI domain in solution-based assays. In this case, the apparent affinity (*K*_*D*_) of hCD11bNb1 was 1 to 6 nM. By contrast, the cell-expressed α_M_β_2_ responded to hCD11bNb1 by increased iC3b∗ binding. One of the major differences between the fluid phase assays with pure components and those involving the cell-expressed α_M_β_2_ was the presence of the cell membrane in the latter experiments. Adair *et al.* (34) previously reported a low-resolution EM structure of the α_M_β_2_ with an HP almost parallel to cell membrane, extending the bent form of the integrin sufficiently at least for smaller ligand to gain access to the α_M_ MIDAS. Modeling of the bent conformation of α_M_β_2_ based on the structure of the α_x_β_2_ ectodomain (2, 3) also supports that the αI domain in bent-closed α_M_β_2_ must be close to the cell membrane (Fig. 6*K*). This membrane proximity may contribute to the unexpected effect of the Nb on cell-bound α_M_β_2_. Binding of the Nb possibly promotes a transition to a more extended conformation (Figs. 1*A* and 6*K*). If this influence of the Nb affects a rate-limiting step with respect to presenting a high-affinity binding site for iC3b, it may explain the agonist behavior of the Nb. This would also agree with the somewhat higher dosage requirement for inducing the agonistic effect (*i.e.*, ∼75 nM) compared with expectations from the formal affinity of hCD11bNb1 for isolated HP (*K*_*D*_ ∼1 nM). In the solution-based protein interaction assays with our α_M_β_2_ HP, this putative membrane-dependent unbending effect on α_M_β_2_ conformation is, of course, not a factor. Here, hCD11bNb1 may either be neutral or act as a weak stabilizer of the α_M_I closed conformation, which together with steric hindrance exerted on the C3d or the iC3b thioester domain explains the observed blocking of iC3b and C3d binding. Nevertheless, if induction of α_M_β_2_ unbending accounts for the agonistic effect of hCD11bNb1, it is surprising that the effect also manifests itself in the presence of Mn^2+^, which conformationally activates integrins. However, in agreement with our proposal, the influence of the Mn^2+^ is mainly through binding to a metal-binding site adjacent to the βI MIDAS, and occupation of this site with Mn^2+^ may not lead to a fully extended integrin unless high concentrations of ligand are present as was noted for α_L_β_2_ (35). Hence, in the cell adhesion assays, repulsion from the membrane may still be able to promote a transition to the high-affinity state of α_M_β_2_.

Despite the possible Nb-induced conformational changes in cell-bound α_M_β_2_, our comparison of crystal structures still predicts that binding of iC3b and hCD11bNb1 is mutually exclusive (Fig. 3*E*). The discrepancy between this prediction and the cell-based assay suggests that iC3b binding to the receptor is more dynamic than apparent from our crystal structure of the C3d–αI core complex (8). Considering that the overlap between the Nb and the iC3b thioester domain only involves a few residues, it is feasible that this overlap can be reduced if the iC3b thioester domain undergoes small internal conformational changes in the region involved in the overlap or the two αI binders readjust slightly relative to each other. In the light of the well-established conformational dynamics of β_2_-integrin receptors, it is plausible that the interaction of iC3b and hCD11bNb1 with the αI domain could be somewhat dynamic on the cell-bound receptor compared with our crystal structures of hCD11bNb1–α_M_β_2_ and C3d–αI domains (8). Hence, complexes where the predicted overlap is reduced or eliminated may actually occur on α_M_β_2_-presenting cells. This agrees with that we and others have demonstrated that there are additional interactions outside the core complex (11) that may support a spectrum of conformations of the iC3b–α_M_β_2_ complex rather than a single rigid complex as captured in the structure of the core complex (8).

## Discussion

### The structure of α_M_β_2_ in the closed conformation

Here, we present the first atomic structure of α_M_β_2_ featuring its HP in the closed conformation characterized by the proximity of the β_2_ hybrid domains to the α_M_ β-propeller and the approach of the β_2_ PSI and I-EGF1 domains to the α_M_ thigh domain. Except for the αI domain, this conformation is strikingly similar to closed conformations known from structures of α_X_β_2_ and α_L_β_2_ (2, 4). Our structure of the closed conformation of α_M_β_2_ is the first step toward establishing the mechanism of outside–in signaling in this receptor. Strikingly, structures of β_2_-integrins in the open-extended conformation of high-ligand affinity are still lacking although low-resolution negative-stain EM micrographs confirm the presence of the open conformation (2, 11). Comprehensive prior studies of other integrins, such as α_v_β_3_ lacking an αI domain, has defined in details the conformational rearrangements occurring in the βI domain upon binding of an external ligand to the βI domain. Ligand binding and modulation of the metal-binding sites in the βI domain propagates into swing out of the hybrid domain and presumably favors extension of the β_2_ subunits and its associated α-subunit and culminates in outside–in signaling (36). The β_2_ integrins, including α_M_β_2_, are expected to react in a similar manner to α-subunit ligand binding and binding of the internal ligand to the βI domain.

### The αI domain in α_M_β_2_

Our comparison of the α_M_β_2_ structure with structures of α_L_β_2_ and α_X_β_2_ demonstrated a unique orientation of the αI domain relative to the platform in the closed conformation. Furthermore, two crystal structures of a bent α_X_β_2_ in which the internal ligand interacts with the βI MIDAS site demonstrated a slight variation in the orientation of the αI domain adopting the open conformation (3). The internal ligand region in these two structures is highly extended, and overall, these structures indicate that the distance between the MIDAS sites in the αI and βI domains as well as the orientation of the αI domain relative to the platform is not necessarily fixed (3). In crystal structures of β_2_ integrins, including our α_M_β_2_ structure, lattice packing appears to play a major role in stabilizing the position of the αI domain. Thus, it is possible that the αI domain is never locked relative to the platform in a cell-bound β_2_ integrin. One striking example supporting this notion is the complex between α_x_β_2_ and its iC3b ligand, where negative-stain 2D classes revealed two opposite orientations of the ligand compared with the platform. This implies that in the ligand-bound state, two orientations of the αI domain differing by up to 180° were present in the sample (37). Nevertheless, other studies of α_M_β_2_ by negative-stain EM featured a more defined orientation of the αI domain relative to the platform (34). Our own 3D reconstructions of the α_M_β_2_ HP also offered evidence that the αI domain is at least somewhat restricted with respect to rotation relative to the platform (11). However, because of the resolution in negative stain and the roughly spherical shape of the αI domain, it is difficult to quantitate the variability in domain orientation from such data.

At present, the only ligand-bound structures involving αI domains from β2 integrins are our α_M_β_2_ αI–C3d complex (8) and the complexes of α_L_β_2_ αI with ICAM-1/3/5 (9, 38, 39). In addition, a model for the α_M_β_2_ αI–GP1bαN complex based on NMR restraints and the crystal structure of the murine glycoprotein Ibα N-terminal domain featured the interaction of an aspartate from a ligand α-helix (40). Detailed structures of multiple integrin–ligand complexes with intact ectodomain or their HP fragments are required to establish the relationship between high-affinity ligand binding, the conformational freedom of the αI domain, and the structural events underlying outside–in signaling in α_M_β_2_ and other β_2_ integrin–ligand complexes. To avoid crystal-packing effects on the αI location and the conformation of the rest of the receptor, single-particle analysis by cryo-EM is likely to be the best approach for establishing the detailed molecular mechanism of outside–in and inside–out signaling of the β_2_-integrins.

A very recent crystal structure of iC3b in complex with the αI domain was reported, which confirmed the core interaction between αI and the iC3b thioester domain (41) previously captured in the αI–C3d complex (8). Two different crystal-packing interactions between the αI domain and domains in iC3b far from the thioester domain were suggested to mirror cell-bound α_M_β_2_ interaction with iC3b on an opsonized surface. Additional experimental evidence is needed to confirm the suggested *in vivo* relevance of these αI interactions with regions outside the iC3b thioester domains.

### Function-modulating molecules targeting α_M_β_2_

*In vivo* studies leave no doubt about the importance of the α_M_β_2_ as a protective agent against infection (42) and as an aggravating factor in diseases with a poorly regulated inflammatory response, for instance, as observed in animal models of multiple sclerosis and Alzheimer’s disease (43). For multiple sclerosis, there is evidence from the pharmacological mode of action of drugs and animal models that α_M_β_2_ may also play in this case an aggravating role, at least in the relapsing-remitting form of the disease (44, 45). With respect to stroke, blocking of α_M_β_2_ by the use of the hookworm-derived neutrophil inhibitory factor improved the outcome in animal models (46). Later trials in humans were however compromised, by pre-existing antibodies to this parasite protein. SLE is an autoimmune disease where complement plays a central role. It is a long-standing observation that α_M_β_2_ expression increases in neutrophils and scales with the severity of the disease (47). α_M_β_2_ has recently been implicated in SLE and lupus nephritis, a kidney disease that is a common complication of SLE (48). Three missense mutations in the gene coding for α_M_ have shown a strong association with both SLE and lupus nephritis in genome-wide association studies (25, 49, 50). The negative impact on α_M_β_2_ function and strong association with SLE for the α_M_ R77H variant has been difficult to explain, but our structural data now suggest that the arginine to histidine mutation could affect the structural dynamics of the ectodomain through long-range effects on conformation and dynamic properties of residues in the α_M_–β_2_ subunit interface.

The examples of aforementioned α_M_β_2_-linked diseases demonstrate that pharmacological regulation of α_M_β_2_ activity is clinically relevant. The complications with respect to therapeutic modulation of the receptor and the repertoire of natural and man-made molecules targeting α_M_β_2_ has recently been extensively reviewed (51). Currently, the most advanced drug candidate is the α_M_β_2_ agonist leukadherin-1 (LA-1), a small molecule that stimulates leukocyte α_M_β_2_ interaction with ICAM-1 and iC3b-presenting cells (52). Mechanistically, LA-1 suppresses leukocyte infiltration into tissues by increasing α_M_/CD11b-dependent cell adhesion to ICAM-1 on the endothelium, preventing subsequent extravasation (53, 54). Modeling suggests that LA-1 binds at the interface between the αI and βI domains and involves the C-terminal end of the αI α7 helix carrying the internal ligand. Such a binding pocket is difficult to reconcile with considerable rotational freedom of the αI domain in the ligand-bound state, so in LA-1-bound α_M_β_2_, the αI domain may have significantly less rotational freedom compared with α_M_β_2_ not binding this small-molecule drug. Our crystal structure provides a valuable scaffold for accurate modeling of α_M_β_2_ complexes with existing and future function-modulating molecules.

### The mechanism and application of the hCD11bNb1 Nb

In our report, we characterize an Nb-based α_M_β_2_ agonist with previously unappreciated mode of action. With an epitope on the αI domain, hCD11bNb1 represents a highly specific reagent compared with conventional monoclonal antibodies, which stimulate ligand interaction to α_M_β_2_ by manipulating the conformation of the β_2_ subunit (55). The Nb stimulated binding of iC3b to cell-bound α_M_β_2_ similar to the agonist LA-1, but with an epitope quite far from the putative LA-1-binding site at the α_M_–β_2_ interface. The mechanism of hCD11bNb1 stimulation of iC3b binding to α_M_β_2_ on cells appears to be unique and complex in the light of the inhibition of iC3b–α_M_β_2_ HP and C3d–αI interaction observed in binding experiments with the pure components.

In general, a Nb is a versatile module that is easily humanized and targeted to specific tissues and cell types by fusion to other proteins. Fusion may also increase the short circulation time of unmodified Nbs (reviewed in Ref. (56)). Animal experiments could investigate the *in vivo* utility of properly modified hCD11bNb1 as a highly specific α_M_β_2_ agonist, but such studies are complicated by the lack of crossreactivity with the murine αI domain. Another major complication with respect to the *in vivo* effects of our Nb is the large number of proteins reported to interact with the αI domain besides iC3b (51), with ICAM-1, fibrinogen, RAGE, JAM-C, and glycoprotein bα as prominent examples. For other ligands, steric hindrance exerted by hCD11bNb1 could be larger than for the C3d/iC3b–αI interactions investigated here. In such cases, rather than functioning as an agonist, the Nb may function as an antagonist. In contrast, if steric hindrance with the Nb does not occur for other α_M_β_2_–ligand pairs, an even stronger stimulation of ligand binding by hCD11bNb1 may be experienced.

## Experimental procedures

### Nb selection

The hCD11bNb1 Nb was selected as previously described (29). Briefly, a *Lama glama* was immunized with the α_M_β_2_ HP by Capralogics (www.capralogics.com), and the peripheral blood lymphocytes were isolated from a blood sample. The RNA was purified from these lymphocytes and used to prepare a complementary DNA library. The region corresponding to the variable domain of heavy-chain only antibodies were cloned into a phagemid vector by PCR, and phage display was used to select Nbs specific toward the αI domain of α_M_β_2_. *Escherichia coli* TG1 cells harboring the phagemid vectors were coinfected with the VCMS13 helper phages and grown for 16 h at 30 °C to generate Nb-presenting phages. Meanwhile, one well in a microtiter plate was coated with 1 μg of α_M_β_2_ αI domain in 100 μl PBS and 3 mM MgCl_2_. The well of the microtiter plate was subsequently blocked by addition of PBS and 3 mM MgCl_2_ supplemented with 2% (w/w) bovine serum albumin (BSA). Next, 3 × 10^12^ Nb-presenting M13 phages were added to the well, and the plate was incubated for 1 h at room temperature to allow binding of phages to the αI domain. Next, the well was washed 15 times in PBS, 3 mM MgCl_2_, 0.1% Tween-20, and 15 times in PBS and 3 mM MgCl_2_ to remove unbound phages. The αI domain–binding phages were liberated through competitive elution by addition of a 100-fold molar excess, to α_M_β_2_ αI domain, of recombinant C3d in PBS and 3 mM MgCl_2_. The eluted phages were amplified in the ER2748/TG1 strain *E. coli* and provided the basis for the second round of selection, performed similarly, however only using 0.1 μg α_M_β_2_ αI domain for coating. ELISA was used to identify Nbs binding the α_M_β_2_ αI domain. To this end, an ELISA plate was coated with 100 μl of 0.1 μg/ml α_M_β_2_ αI domain in PBS and 3 mM MgCl_2_. Meanwhile, in a 96-well format, single phage–infected colonies were inoculated LB and grown at 37 °C for 6 h followed by induction of Nb expression by addition of isopropyl β-d-1-thiogalactopyranoside to a final concentration of 0.8 mM. The cells were grown for 16 h at 30 °C, then pelleted, and the Nb-enriched supernatant was transferred to the ELISA plate. The plate was incubated for 1 h followed by six washes in PBS, 3 mM MgCl_2_, and 0.1% Tween-20. Then, 1:10,000 diluted E-tag-horseradish peroxidase antibody (Bethyl) was added, and the plate was incubated for 1 h. The plate was washed and developed using 3,3′,5,5′-tetramethylbenzidine, until a clear signal was obtained. About 1 M HCl was added to stop further development, and the absorbance at 450 nm was measured. Unique Nbs were identified by sequencing and subsequently cloned into the bacterial expression vector pET-22b(+).

### Protein production

α_M_β_2_ HP was produced as previously described (11). In short, the supernatant of stable human embryonic kidney 293S cells expressing α_M_β_2_ HP was recovered and purified by immobilized ion-affinity chromatography using a 5 ml HisTrap Excel column (GE Healthcare). The protein was subsequently applied to a 1 ml StrepTactin column (GE Healthcare) yielding pure α_M_β_2_ HP. The affinity tags and coiled coil domains were removed by addition of the 3C protease, and a final polishing step was performed using SEC into 20 mM Hepes (pH 7.5), 150 mM NaCl, 5 mM MgCl_2_, and 1 mM CaCl_2_. Recombinant αI domain was purified as described (11).

C3 was purified and cleaved to C3b as described (29). C3b was cleaved to iC3b by addition of 1% (w/w) factor H (Complement Tech) and 0.2% (w/w) factor I (Complement Tech), and the reaction was incubated for 16 h at 4 °C. The cleavage was assessed by SDS-PAGE and stopped by addition of 0.5 mM Pefabloc SC. To remove C3c or C3b, the sample was loaded on a 1 ml MonoQ column (GE Healthcare) equilibrated in 20 mM Hepes (pH 7.5) and 200 mM NaCl. The protein was eluted by a 30 ml linear gradient from 200 to 350 mM NaCl. C3d was purified as described (11). The hCD11bNb1 point mutants I29A, N30A, F32A, F54A, and N101A were generated by site-directed mutagenesis using the QuickChange Lightning kit (Agilent). hCD11bNb1, hCD11bNb1 mutants, and avi-tagged hCD11bNb1 was purified and generated as described for hC3Nb1 and avi-tagged hC3Nb1 (29). Endotoxin removal from Nbs used for flow cytometry was performed as described (57). Endotoxin levels were quantified using LAL chromogenic endotoxin quantification kit (Thermo Fisher Scientific) performed as described by the manufacturer. Nbs with endotoxin levels below 2 EU/mg Nb were considered to be endotoxin free.

### SEC assays

For analysis of hCD11bNb1: α_M_β_2_–αI interaction, 40 μg α_M_β_2_ αI domain was incubated in the presence or the absence of a 1.5-fold molar excess of hCD11bNb1 in 20 mM Hepes (pH 7.5), and 150 mM NaCl. The mix was incubated for 30 min on ice and then applied to a 24 ml Superdex 75 increase (GE Healthcare) column equilibrated in 20 mM Hepes (pH 7.5) and 150 mM NaCl. For analysis of the inhibition, 170 μg αI domain and an equimolar amount of C3d were incubated for >5 min on ice in the presence or the absence of a twofold molar excess of hCD11bNb1 in a reaction buffer containing 20 mM Hepes (pH 7.5), 150 mM NaCl, and 2 mM MgCl_2_. The mix was next applied to a 24 ml Superdex 75 increase column equilibrated in 20 mM Hepes (pH 7.5), 150 mM NaCl, and 2 mM MgCl_2_.

### Structure determination

Crystals of hCD11bNb1 were grown by vapor diffusion at 4 °C by mixing an hCD11bNb1 solution at 35 mg/ml 1:1 with reservoir solution containing 1.5 M AmSO_4_ and 0.1 M Bis–Tris (pH 6.5). The crystals were soaked in reservoir solution supplemented with 30% glycerol before being flash frozen in liquid nitrogen. The data were collected at BioMAX (MAX IV) at 100 K and processed with XDS (58). A search model was prepared for molecular replacement using Phenix.sculpt (59), and the structure was solved with Phaser (60). Missing residues and side chains were built using Coot (61). In an iterative manner, the structure was rebuilt in Coot and refined with Phenix.refine using positional refinement, individual *B*-factors, and TLS groups. In the last round of refinement, anisotropic *B*-factors were refined for the sulfur atoms.

Prior to crystallization of the Nb complex, α_M_β_2_ HP in 20 mM Hepes (pH 7.5), 150 mM NaCl, 5 mM MgCl_2_, and 1 mM CaCl_2_ was mixed with a 1.5-fold molar excess of hCD11bNb1 to a final complex concentration of 9 mg/ml. Crystals were grown at 19 °C by vapor diffusion in sitting drops made by mixing the complex in a 1:1 ratio with reservoir containing 1.25 M sodium malonate, 76 mM Hepes (pH 8.0), 24 mM Hepes (pH 6.5), and 0.5% Jeffamine ED2001 (pH 7.0). The crystals were soaked in a saturated sodium malonate solution before being flash frozen in liquid nitrogen. The data were collected at BioMAX at 100 K and processed with XDS (58). The structure was determined using the coordinates of the β-propeller from α_X_β_2_ (Protein Data Bank [PDB] entry: 4NEH) and βI domain of α_L_β_2_ (PDB entry: 5E6S) in Phaser (60). The remaining domains were placed manually in Coot (61). The resulting model was refined with rigid body refinement in Phenix.refine (59). At this stage, it had become apparent that the data suffered from anisotropic diffraction. The data were therefore scaled anisotropically using the STARANISO server (24). Following this, the structure was manually rebuilt in Coot and refined with Phenix.refine using positional refinement, grouped *B*-factors, and TLS groups in an iterative manner. In the final round of refinement, individual *B*-factor refinement was conducted.

### BLI

All BLI experiments were performed on an Octet Red96 (ForteBio) at 30 °C and shaking at 1000 rpm. The running and wash buffer is 20 mM Hepes (pH 7.5), 150 mM NaCl, 5 mM MgCl_2_, and 1 mM CaCl_2_ unless otherwise stated. For assessing the binding of hCD11bNb1 to α_M_β_2_ HP, anti–penta-HIS sensors (ForteBio) were first washed for 2 min, followed by a 5 min loading step where hCD11bNb1 at 5 μg/ml was loaded on the sensors. Subsequently, the sensors were washed for 30 s and then baselined for 2 min. Association to α_M_β_2_ HP (50, 25, 12.5, 6.25, and 0 nM) was followed for 3 min, followed by a 5 min dissociation step. The binding assay with the α_M_β_2_ αI domain was performed in the same manner, except that the association was followed for 300 s, and that the concentrations used were 25, 12.5, 6.25, 3.135, 1.6, 0.8, and 0 nM. All experiments were performed in triplicates. The 0 nM measurements were subtracted from all data series before fitting to a 1:1 Langmuir binding model. The association was modeled as: *R*(*t*) = *R*_max_([α_M_β_2_]/([α_M_β_2_] + *K*_*D*_)(1−exp(−t·(*k*_on_·[α_M_β_2_] − *k*_off_))), *K*_*D*_ = *k*_on_/*k*_off_, and the dissociation was modeled as a first-order exponential decay, *R*(*t*) = *R*(300)·exp(−k_*o*ff_(*t* − 300 s)).

For the competition assay assessing the ability of different hCD11bNb1 mutants to compete with WT hCD11bNb1 for α_M_β_2_ binding, the streptavidin biosensors (ForteBio) were first washed for 2 min in running buffer supplemented with 1 mg/ml BSA, before biotinylated avi-tagged hCD11bNb1 at 5 μg/ml were loaded on the sensors for 5 min. The sensors were then washed for 2 min in running buffer supplemented with 1 mg/ml BSA, before being baselined for 2 min. Thereafter, association between 20 nM α_M_β_2_ HP alone or 20 nM α_M_β_2_ HP preincubated with 10-fold molar excess of hCD11bNb1 mutants I29A, N30A, F32A, F54A, N101A, or WT hCD11bNb1 was followed for 5 min. Subsequently, the dissociation was followed for 5 min.

For analysis of the interaction between hCD11bNb1 and murine α_M_β_2_ αI, anti–penta-HIS sensors were washed for 5 min in 20 mM Hepes (pH 7.5) and 150 mM NaCl. The hCD11bNb1 at 5 μg/ml was loaded onto the sensors, followed by a 2 min wash step and a 2 min baselined step. The association of hCD11bNb1 to murine αI (50, 25, 12.5, 6.25, 3.135, and 0 nM) or 50 nM human αI was followed for 5 min followed by a 5 min dissociation step. The 0 nM measurement was subtracted from all data series. This experiment was performed in duplicates.

### SPR

The SPR experiment was performed on a Biacore T200 (GE Healthcare) instrument as described (11). The system was equilibrated in running buffer 20 mM Hepes (pH 7.5), 150 mM NaCl, 5 mM MgCl_2_, and 1 mM CaCl_2_. Streptavidin was immobilized on a CMD500M chip (XanTec bioanalytics GmbH) to 200 response units. Next, biotinylated iC3b was injected on one flow cell in excess, saturating the chip surface. For competition experiment, α_M_β_2_ at 1.25 to 100 nM was injected in either the presence or the absence of a 1.5-fold molar excess of hCD11bNb1. Upon the competition experiment, 50 mM EDTA, 1 M NaCl, and 100 mM Hepes (pH 7.5) were injected over the chip to regenerate the surface.

### Mass photometry

The measurements were performed on glass coverslips and recorded on a mass photometer (MP_TWO_; Refeyn Ltd) (32) for 60 to 120 s. Each measurement was repeated at least twice. The α_M_β_2_ HP and iC3b were diluted immediately prior to measurements in 20 mM Hepes (pH 7.5), 150 mM NaCl, 5 mM MgCl_2_, and 1 mM CaCl_2_ to a concentration of 10 nM. For 1:1 complex formation, α_M_β_2_ HP and iC3b were mixed in a 1:1 M ratio, whereas α_M_β_2_ HP and hCD11bNb1 were mixed in a 1:10 M ratio. The influence of hCD11bNb1 on α_M_β_2_ HP–iC3b complex formation was measured using molar ratios of hCD11bNb1 to α_M_β_2_ HP (0.49, 0.74, 1.1, 1.6, 2.5, 5, and 10). The recorded videos were analyzed using DiscoverMP (Refeyn Ltd; version 2.5.0) to quantify protein-binding events. The molecular weight was obtained by contrast comparison with known mass standard calibrants measured on the same day.

### Cell-expressed α_M_β_2_–ligand interaction with iC3b

The binding of iC3b by cell-expressed α_M_β_2_ was investigated by use of K562 cells with a recombinant expression of α_M_β_2_, or, as a control, α_X_β_2_. For binding under conditions approaching equilibrium, α_M_β_2_/K562 cells were cultured and treated with fluorescence-conjugated iC3b as described (11). Briefly, the cells were kept in buffer with 1 mM Ca^2+^ and 1 mM Mg^2+^ or as further supplemented with 1 mM Mn^2+^ to activate integrin ligand binding. Following 45 min of incubation with 10 μg/ml Alexa Fluor 488-conjugated (iC3b∗) together with 0, 1, 5, or 10 μg/ml of either hCD11bNb1 or an unrelated control (Ctrl) Nb specific for complement C4 called Nb10 (56) kindly supplied by Alessandra Zarantonello. Next, the cells were briefly rinsed, followed by fixation in PBS with 0.99% (v/v) formaldehyde. As a control experiment, the well-characterized function-blocking monoclonal antibody to α_M_ ICRF44 (62) (Sigma–Aldrich) was also applied at 10 μg/ml. The MFI of iC3b∗-bound cells was determined in a NovoCyte Flow Cytometer (Agilent Technologies) by subtracting the (autofluorescence) MFI for cells with no addition of iC3b∗ (ΔMFI).

To investigate the influence of hCD11bNb1 under cell adhesion with a mimetic of the shear stress–influencing cells under physiological conditions, adhesion of α_M_β_2_/K562 or α_X_β_2_/K562 cells was tested in a centrifugation-based assay described earlier (63). Briefly, V-shaped microtiter wells were coated with 1 μg/ml iC3b (A115; Complement Tech) or not coated as reference, and blocked in PBS with 0.05% (v/v) Tween-20. Cells were applied either in buffer with Ca^2+^ and Mg^2+^ or with a further addition of 1 mM Mn^2+^ to activate integrin ligand binding. Nbs were added in concentrations of 0, 1, 5, or 10 μg/ml for either buffer condition. Following incubation for 10 min at 37 °C, the cells were centrifuged at 10*g* for 5 min and read in a fluorescence plate reader. The centrifugation and plate reading were repeated at 30*g*, 50*g*, and 100*g*.

## Data availability

Coordinates and structure factor for the hCD11bNb–α_M_β_2_ complex and hCD11bNb1 are available at the PDB as entries 7P2D and 7NP9.

## Supporting information

This article contains supporting information.

## Conflict of interest

The authors declare that they have no conflicts of interest with the contents of this article.

## Supporting information

Supportinfo
