# Supplementary material for: Structural insights into the function-modulating effects of nanobody binding to the integrin receptor αMβ2
Source: J Biol Chem. 2022 Jun 20;298(8):102168. doi: 10.1016/j.jbc.2022.102168 (PMC9287160; doi:10.1016/j.jbc.2022.102168)
Supplement: Supportinfo [file mmc1.docx]

## Supporting information


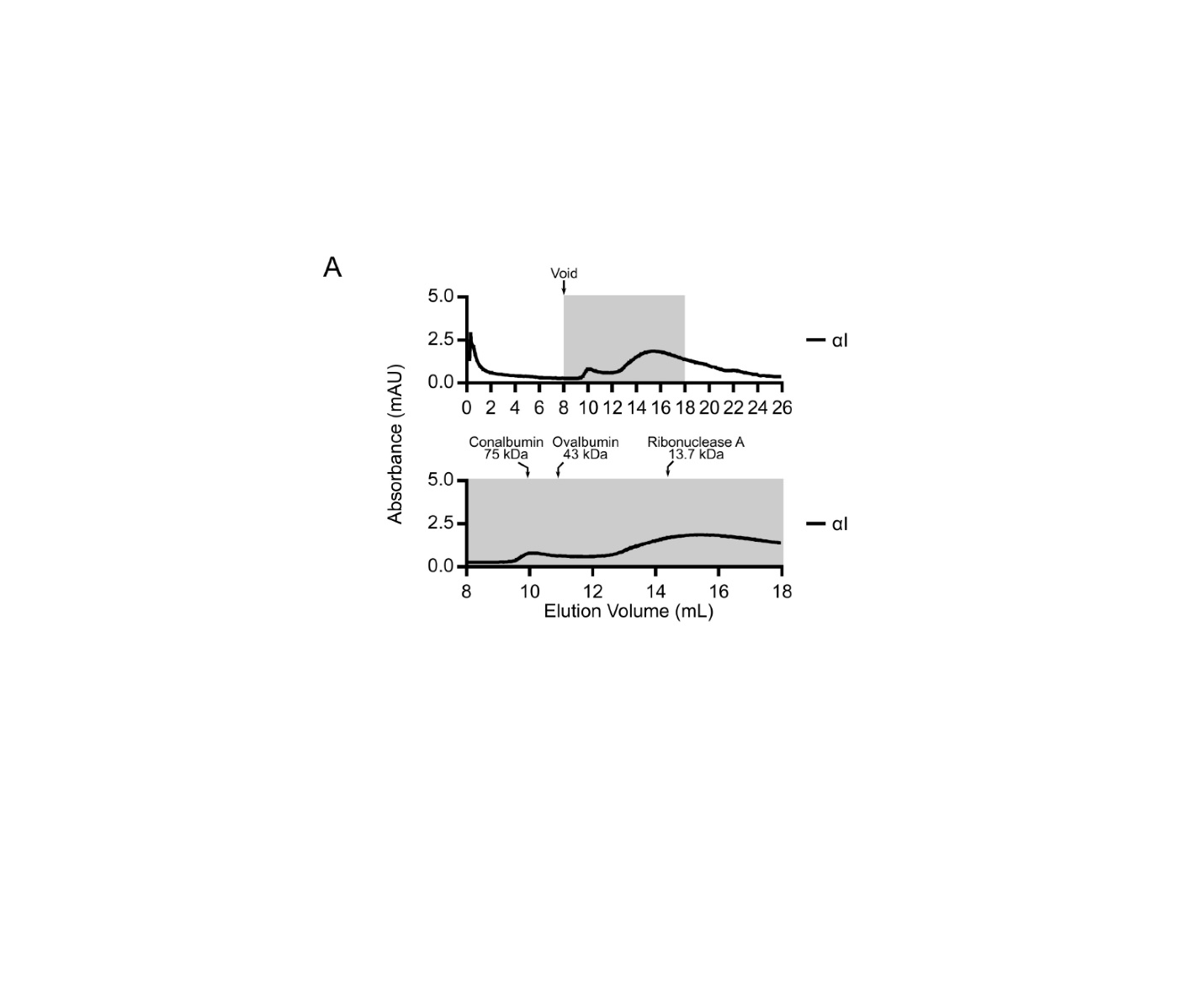


**Figure S1: Elution profile of α_M_β_2_ αI in the presence of Mg^2+^ ions.** SEC analysis of αI in presence of magnesium ions results in a poorly defined elution profile likely to be caused by unspecific interactions of the αI domain with the resin. The magnified view of the shaded area in the lower panel can be compared directly with the elution profiles in figure 1.


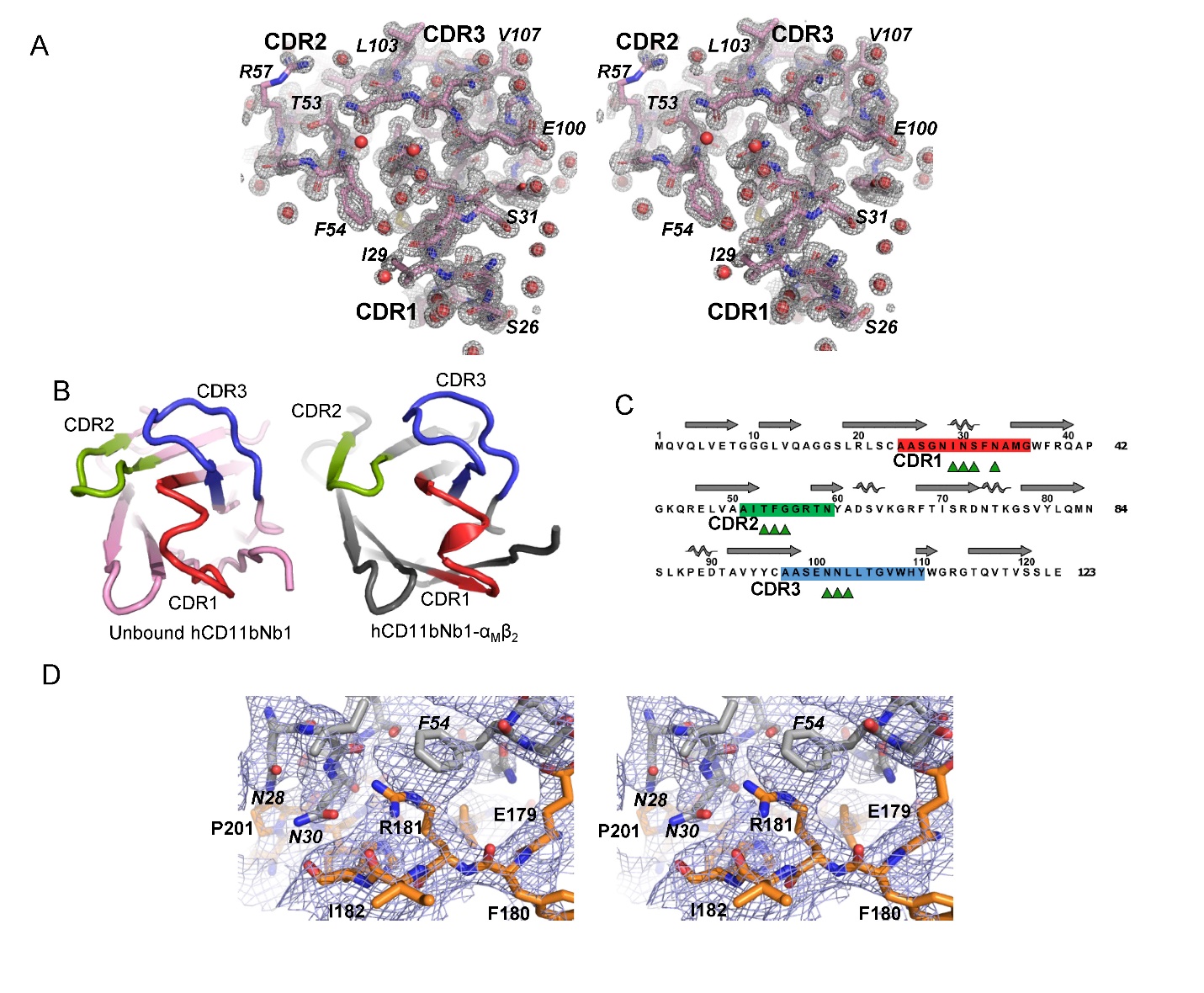


**Figure S2. Details of the structure determination of hCD11bNb1 and its α_M_β_2_** **complex.** (A) Stereo view of an omit 2mF_o_-DF_c_ electron density around complementarity determining regions contoured at 1.5 σ for the 1.14 Å resolution structure of the unbound nanobody. Residues 27-30, 53-57 and 100-103 in the CDRs were omitted for map calculation. (B) End view of the three CDRs demonstrating limited conformational changes upon α_M_ binding. (C) Secondary structure of hCD11bNb1 and definition of the CDRs. Triangles mark residues in the nanobody paratope contacting the α_M_ subunit. (D) Stereo view of an omit 2mF_o_-DF_c_ electron density for the hCD11bNb1-α_M_ interface contoured at 1.0 σ. Labels for residues in the nanobody are italicized. Residues 28-32, 53-55 and 101-103 in hCD11bNb1 and residues 200-206, 179, 181, 183 in α_M_ were omitted for map calculation.


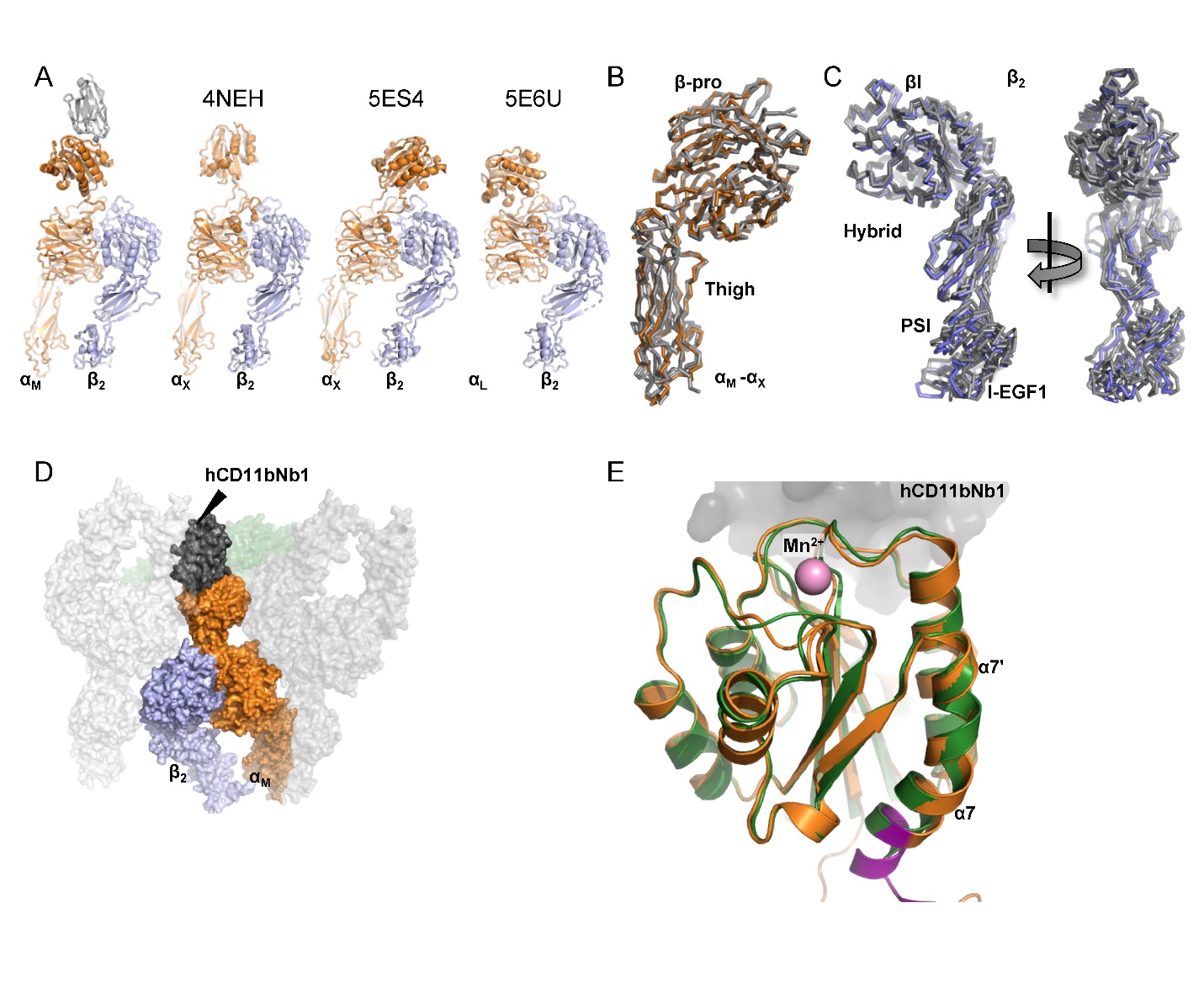


**Figure S3. Comparison of α_M_β_2_** **with known structures of β_2_-integrins.** (A) A line-up of structures of the headpiece fragments (α_M_β_2_ and α_L_β_2_) and the headpiece moiety of two α_x_β_2_ ectodomain structures illustrates their strikingly similar overall conformations when aligned on their βI domains. The PDB entry is listed above each structure. (B) Alignment of α_M_ and α_x_ on their β-propeller domains demonstrates a conserved orientation of their Thigh domains. Compared to structures of a_X_β_2_ (entries 4NEH and 5ES4) the Thigh domain is rotated 4-13° and translated less than 2 Å. (C) As in panel B with the β_2_ chains aligned on the βI and Hybrid domains. Compared to structures of a_X_β_2_ (entries 4NEH and 5ES4) and a_L_β_2_ (5E6U), the PSI/EGF1 domains are rotated 6-18° and translated less than 4 Å. (D) Crystal packing for α_M_β_2_-hCD11bNb1 complex showing how both the nanobody and the αI domain form contacts to three other complexes in the lattice. (E) Comparison of the hCD11Nb1-bound αI domain (orange cartoon but with the internal ligand region in magenta) and the canonical closed conformation of the isolated αI domain in complex with a Mn^2+^ ion in PDB entry 1JLM (green cartoon).
